# Discovery of new mutually orthogonal bioorthogonal cycloaddition pairs through computational screening†
†Electronic supplementary information (ESI) available: Experimental and computational details. See DOI: 10.1039/c5sc03259h


**DOI:** 10.1039/C5SC03259H

**Published:** 2015-11-11

**Authors:** Maruthi Kumar Narayanam, Yong Liang, K. N. Houk, Jennifer M. Murphy

**Affiliations:** a Crump Institute for Molecular Imaging , David Geffen School of Medicine , University of California , Los Angeles , California 90095 , USA . Email: jmmurphy@mednet.ucla.edu; b Department of Chemistry and Biochemistry , University of California , Los Angeles , California 90095 , USA . Email: houk@chem.ucla.edu; c Department of Chemical and Biomolecular Engineering , University of California , Los Angeles , California 90095 , USA

## Abstract

Density functional theory (DFT) calculations and experiments in tandem led to discoveries of new reactivities and selectivities involving bioorthogonal sydnone cycloadditions. Dibenzocyclooctyne derivatives (DIBAC and BARAC) were identified to be especially reactive dipolarophiles, which undergo the (3 + 2) cycloadditions with *N*-phenyl sydnone with the rate constant of up to 1.46 M^–1^ s^–1^. Most significantly, the sydnone-dibenzocyclooctyne and norbornene-tetrazine cycloadditions were predicted to be mutually orthogonal. This was validated experimentally and used for highly selective fluorescence labeling of two proteins simultaneously.

## Abstract 2

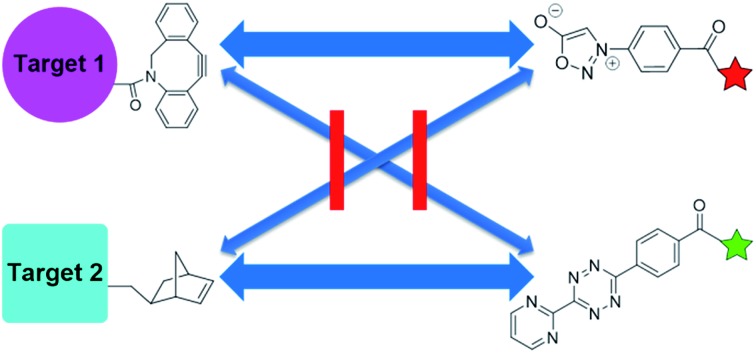
The sydnone-dibenzocyclooctyne and norbornene-tetrazine cycloadditions are both bioorthogonal and mutually orthogonal, used for simultaneous labeling of two targets.

## Introduction

The interrogation of the molecular details of biological processes in living systems demands exquisite selectivity combined with high reactivity. Despite many challenges, the design and tuning of reaction partners has provided a myriad of bioorthogonal chemical reactions, which have had a major impact on the study of many biological processes *in vivo*.^1^ The discovery of useful bioorthogonal reactions is generally empirical. The modified Staudinger reaction, reported by Bertozzi in 2000,^2^ involves organic azides and triaryl phosphines, but is somewhat slow (typical second-order rate constant of 2 × 10^–3^ M^–1^ s^–1^), necessitating the use of high concentrations of phosphine reagents. A remarkable advance in this field was Bertozzi *et al.*'s azide-cyclooctyne (3 + 2) cycloaddition that has enabled cellular component labeling in living systems.^3^ Stimulated by this distortion-accelerated process, nearly all recent additions to the bioorthogonal reaction repertoire are cycloadditions,^4^ especially 1,3-dipolar^5^ and Diels–Alder^6^ reactions. A great deal of progress has been made towards developing new reactions for labeling biomolecules with extraordinarily high rate constants and monitoring multicomponent processes in complex environments.^7^

**Scheme 1:**
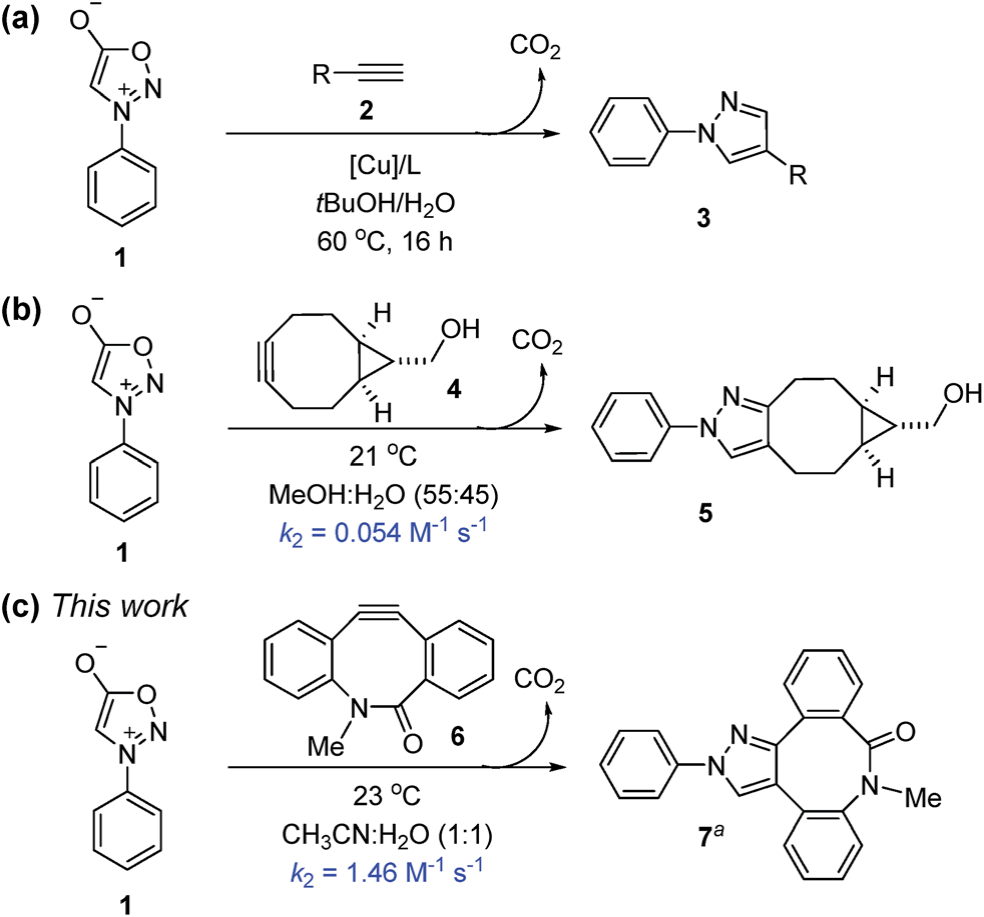
Reactivity of the (3 + 2) cycloadditions involving *N*-phenyl sydnone (^*a*^only one regioisomer is depicted).

First discovered in 1935, sydnones are remarkably stable mesoionic heterocycles that contain an azomethine imine moiety.^8^ In the early 1960s, Huisgen reported that, upon treatment with alkynes at 170 °C, sydnones undergo (3 + 2) cycloadditions to form pyrazoles, *via* a bicyclic intermediate which eliminates carbon dioxide.^9^ Although sydnones have been used for the synthesis of biologically interesting molecules,^10^ the potential of sydnones as bioorthogonal reaction partners was overlooked until recently. Using a high-throughput immunoassay screening, Taran and co-workers discovered that sydnones are biocompatible and undergo 1,3-dipolar cycloadditions with terminal alkynes in the presence of a copper phenanthroline complex to produce 1,4-pyrazoles in up to 99% yield (Scheme 1a).^11^ Chin developed a Cu-free strain-promoted (3 + 2) cycloaddition between *N*-phenyl sydnone and bicyclo-[6.1.0]-nonyne (BCN) at ambient temperature to afford the corresponding pyrazole in 30 minutes, with an isolated yield of 99% (Scheme 1b).^12^ The rate constant in MeOH–H_2_O (55 : 45) was determined to be 0.054 M^–1^ s^–1^ at 21 °C.^13^

These results inspired our investigation into the reactivity of sydnones as 1,3-dipoles for bioorthogonal cycloadditions. Since many strained alkenes and alkynes have been well established as bioorthogonal 2π cycloaddends, it is now efficient to evaluate reactivities towards *N*-phenyl sydnone through computational screening. Here we report the computation-guided discovery of two new bioorthogonal (3 + 2) cycloaddition reactions of *N*-phenyl sydnone with biarylazacyclooctynone (BARAC) and dibenzoazacyclooctyne (DIBAC), which demonstrate significant rate enhancement over the previously reported sydnone-BCN cycloaddition.^12^ In addition, with the aid of DFT calculations, we design and identify two mutually orthogonal reaction pairs, which enable highly selective fluorescence labeling of two proteins simultaneously.

## Results and discussion

**Fig. 1:**
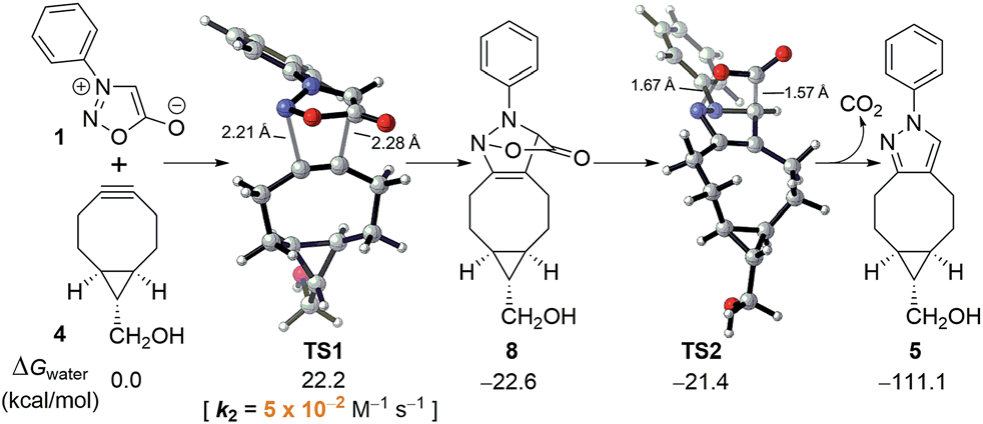
Energetics and transition states for the (3 + 2) cycloaddition of *N*-phenyl sydnone (**1**) with BCN **4** and subsequent CO_2_ release.

To better understand the sydnone-BCN cycloaddition and to accelerate the discovery of new bioorthogonal chemistry involving *N*-phenyl sydnone, DFT calculations were performed at the M06-2X/6-311+G(d,p)//M06-2X/6-31G(d) level of theory.^14^,^15^ We have previously established the reliability of prediction of rates in aqueous solution with this method.^16^ As shown in Fig. 1, the (3 + 2) cycloaddition of sydnone **1** with BCN **4***via* transition state **TS1** requires an activation free energy of 22.2 kcal mol^–1^ in water, and the formation of cycloadduct **8** is exergonic by 22.6 kcal mol^–1^. The conversion of intermediate **8** to final pyrazole product **5** by the release of CO_2_*via* transition state **TS2** has almost no barrier. The overall reaction is strongly exergonic by 111.1 kcal mol^–1^. This indicates that, after the 1,3-dipolar cycloaddition, the subsequent CO_2_ release occurs spontaneously, and that the reaction rate is entirely controlled by the (3 + 2) cycloaddition step.

**Fig. 2:**
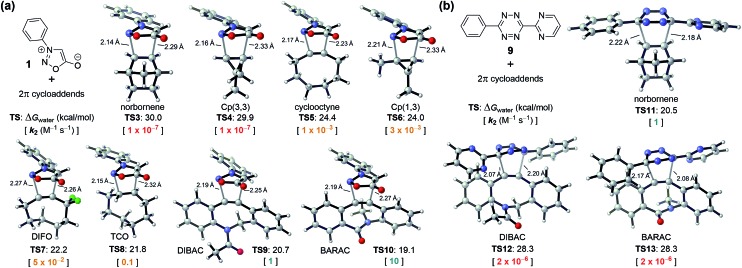
(a) DFT-computed activation free energies for the (3 + 2) cycloadditions of sydnone **1** with eight strained alkenes and alkynes at the CPCM(water)-M06-2X/6-311+G(d,p)//M06-2X/6-31G(d) level of theory and the predicted rate constants in water at 25 °C. (b) DFT-computed activation free energies and predicted rate constants for the (4 + 2) cycloadditions of tetrazine **9** with norbornene, DIBAC, and BARAC.

As shown in Fig. 2a, we located transition-state structures **TS3–10** for eight (3 + 2) cycloadditions of *N*-phenyl sydnone with strained alkenes and alkynes previously used as bioorthogonal reagents. On the basis of the DFT-computed activation free energies (**TS1** and **TS3–10**, Fig. 1 and 2a) and the experimental rate constant of the sydnone-BCN cycloaddition (*k*_2_ = 5 × 10^–2^ M^–1^ s^–1^, Scheme 1b), we predicted a series of rate constants (*k*_2_) of unexplored bioorthogonal sydnone cycloadditions. It was found that the range of reactivities is enormous, with activation free energies varying from 30 to 19 kcal mol^–1^, which correspond to a 10^8^ range in rate constants at room temperature. For cyclooctyne, 1,3-disubstituted cyclopropene (Cp(1,3)), difluorocyclooctyne (DIFO), and *trans*-cyclooctene (TCO) (**TS5–8**), the predicted rate constants range from 10^–3^ to 10^–1^ M^–1^ s^–1^ (shown in yellow, Fig. 2a). These data are very close to the reported rate constant for the sydnone-BCN cycloaddition. This suggests that these reactions may be employed for biomolecular labeling but may not be suitable for *in vivo* applications due to the moderate rate constants. To our delight, calculations predicted that when *N*-phenyl sydnone reacts with DIBAC and BARAC (**TS9–10**), the rate constants will be 1 and 10 M^–1^ s^–1^, respectively (shown in green, Fig. 2a). By contrast, sydnone **1** is predicted to be inert to norbornene and 3,3-disubstituted cyclopropene (Cp(3,3)) (**TS3–4**) under physiological conditions, according to the expected rate constants of 10^–7^ M^–1^ s^–1^ (shown in red, Fig. 2a). This dramatic reactivity difference between dibenzocyclooctyne derivatives (DIBAC and BARAC) and norbornene in the sydnone cycloaddition was then exploited for design of mutually orthogonal bioorthogonal reaction pairs.

Fig. 2b shows that diaryltetrazine **9** reacts smoothly with norbornene (**TS11**). However, the Diels–Alder reactions of DIBAC and BARAC (**TS12–13**) with diaryltetrazine **9** will not occur at room temperature, according to the predicted rate constants of 2 × 10^–6^ M^–1^ s^–1^ (shown in red, Fig. 2b). As a result, the sydnone-dibenzocyclooctyne and norbornene-tetrazine cycloadditions are predicted to be mutually orthogonal.

Our previous computational studies showed that the distortion energy of substrates is crucial to the reactivity of bioorthogonal cycloadditions.^16^,^17^ Usually, the more pre-distorted substrate is more reactive as it takes less energy to reach its transition-state geometry. Dibenzocyclooctyne derivatives (DIBAC and BARAC) have much smaller alkyne bond angles than normal alkynes (*ca.* 153° *versus* 180°),17*a* while norbornene is just pre-distorted by *ca.* 8° as compared to normal alkenes.17*b* Therefore, in sydnone cycloadditions, dibenzocyclooctyne derivatives (DIBAC and BARAC) are significantly more reactive than norbornene. However, DIBAC and BARAC exhibit steric hindrance due to the two aryl hydrogen atoms *ortho* to the alkyne,^16^ so their Diels–Alder reactions with bulky disubstituted tetrazines are dramatically slower than that of norbornene.

To test these predictions, we first measured the experimental rate constant for the 1,3-dipolar cycloaddition of *N*-phenyl sydnone (**1**) with BARAC **6** (for details, see the ESI†). As expected, a significantly enhanced second-order rate constant of 1.46 M^–1^ s^–1^ was obtained for the (3 + 2) cycloaddition in MeCN–H_2_O (1 : 1) at 23 °C (Scheme 1c). This is about 30 times larger than that for the sydnone-BCN cycloaddition.^12^

**Scheme 2:**
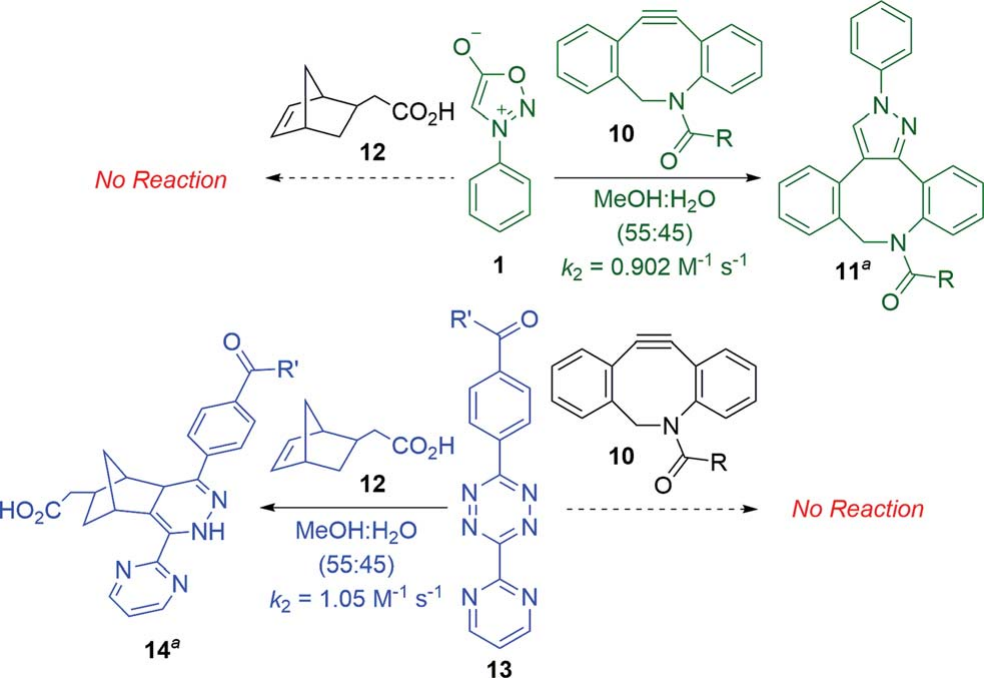
Mutual orthogonality between sydnone-DIBAC and norbornene-tetrazine cycloadditions (^*a*^R = CH_2_CH_2_NH_2_, R′ = NH(CH_2_)_3_NHBoc; only one regioisomer is depicted).

Scheme 2 summarizes experiments to test the predicted mutual orthogonality between sydnone-DIBAC and norbornene-tetrazine cycloadditions. The second-order rate constant for the (3 + 2) cycloaddition of DIBAC **10** with sydnone **1** in MeOH–H_2_O (55 : 45) was determined to be 0.902 M^–1^ s^–1^ (for details, see the ESI†). The Diels–Alder reaction of diaryltetrazine **13** with 5-norbornene-2-acetic acid (**12**) was found to have a *k*_2_ of 1.05 M^–1^ s^–1^ (for details, see the ESI†) in MeOH–H_2_O (55 : 45), which is comparable to rate constants reported in the literature for similar systems.^18^ To test potential cross-reactivity, sydnone **1** and norbornene **12** or diaryltetrazine **13** and DIBAC **10** were incubated in an NMR tube in deuterated methanol and monitored *via* NMR spectroscopy. As predicted, no reaction was observed between either reaction pair at room temperature after 24 hours.

Probing multiple biomolecules, within their native cellular habitat, requires the selective modification of each system with chemical moieties that are compatible with each other. A remarkable expansion of the bioorthogonal toolbox has equipped chemical biologists with a range of selective reactions.^4^,^7^ However, the highly complex nature of biological processes often demands concurrent modification and tracking of multiple targets within the same system. Clever manipulation of these various reactions has enabled the use of sequential click chemistry reactions to tag multiple targets in a single system without perturbations to the system.^19^ Hilderbrand and others have demonstrated the orthogonality between the Diels–Alder tetrazine ligation and the 1,3-dipolar azide cycloaddition to facilitate multicomponent labeling.^20^

**Fig. 3:**
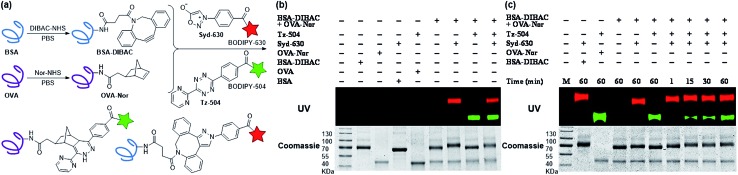
Modifications of protein surfaces *via* bioorthogonal cycloadditions. (a) Dibenzoazacyclooctyne (DIBAC) and 5-norbornene-2-acetic acid (Nor) were appended to BSA and OVA (20 mg mL^–1^ in PBS) respectively, *via* NHS ester-amine coupling conditions (20 mM labeling reagent). The labeled proteins **BSA-DIBAC** and **OVA-Nor** (2 mg mL^–1^) simultaneously react with sydnone-BODIPY (**Syd-630**) and tetrazine-BODIPY (**Tz-504**). (b) Gel analysis of BSA functionalized with DIBAC and OVA functionalized with Nor incubated for 1 h with either **Syd-630**, **Tz-504**, both reagents simultaneously, or no reagent (–). (c) Gel analysis of DIBAC-modified BSA and Nor-modified OVA with both **Syd-630** and **Tz-504** simultaneously for 1–60 min or no reagent (–). The protein loading on the gels was assessed with Coomassie stain.

Leveraging the excellent orthogonality between sydnone-DIBAC and norbornene-tetrazine cycloadditions, we examined the application of this chemistry towards dual fluorescence protein labeling. The DIBAC and norbornene moieties were appended to the surface of two ubiquitous proteins, bovine serum albumin (**BSA**) and ovalbumin (**OVA**) using standard coupling conditions (Fig. 3a). The modified proteins (**BSA-DIBAC** and **OVA-Nor**) were treated with either red fluorescent sydnone-BODIPY630 (**Syd-630**) or green fluorescent tetrazine-BODIPY504 (**Tz-504**) or both and analyzed *via* in gel fluorescence imaging. When the modified proteins were jointly treated with **Syd-630**, only **BSA-DIBAC** was fluorescently labeled (lane 7, Fig. 3b). Likewise, in the presence of **Tz-504**, **OVA-Nor** showed selective labeling without any detection of **BSA-DIBAC** fluorescence labeling (lane 8, Fig. 3b). Co-administration of both fluorophore conjugates in “one pot” resulted in the concurrent labeling of both proteins (lane 9, Fig. 3b). Unmodified proteins **BSA** and **OVA** showed no fluorescence labeling after exposure to **Syd-630** and **Tz-504** (lanes 4 and 5, Fig. 3b), indicating the absence of nonspecific reactivity. In a time-dependent experiment, the protein **BSA-DIBAC** underwent labeling and exhibited fluorescence within 1 min (lane 7, Fig. 3c), whereas the protein **OVA-Nor** exhibited fluorescence labeling after 15 min (lane 8, Fig. 3c). These experiments demonstrate the exquisite specificity of the sydnone and tetrazine imaging agents for DIBAC- and norbornene-modified proteins, respectively, as well as their mutual compatibility within a single environment. Moreover, for the new sydnone-DIBAC cycloaddition, the ability to undergo fluorescence labeling within 1 min using as little as 12 nM protein (with 500 μM dye) is very promising for *in vivo* imaging.

## Conclusions

With the aid of computational screening, we have discovered two rapid cycloadditions between *N*-phenyl sydnone and dibenzocyclooctyne derivatives (DIBAC and BARAC) that proceed in aqueous media, at physiological temperature without the use of a catalyst. The predicted mutually orthogonal sydnone-DIBAC and norbornene-tetrazine cycloaddition pairs have been successfully applied to fluorescence labeling of two proteins simultaneously. DFT calculations shown in Fig. 2a also predicted that sydnone is inert to 3,3-disubstituted cyclopropene. Our previous study has proven that 3,3-disubstituted cyclopropenes react with nitrile imines *via* 1,3-dipolar cycloaddition but not with tetrazines *via* Diels–Alder reaction.19*d* This suggests that precise control over cycloaddition selectivity (dibenzocyclooctynes with sydnones, norbornenes with tetrazines, and 3,3-disubstituted cyclopropenes with nitrile imines) can enable labeling of three targets in a biological environment. We anticipate that these results will complement the expanding toolkit of bioorthogonal reactions and advance the efforts to simultaneously examine multiple processes within a biological system.

## Supplementary Material

Supplementary information

## References

[cit1] Sletten E. M., Bertozzi C. R. (2009). Angew. Chem., Int. Ed..

[cit2] Saxon E., Bertozzi C. R. (2000). Science.

[cit3] Agard N. J., Prescher J. A., Bertozzi C. R. (2004). J. Am. Chem. Soc..

[cit4] Jewett J. C., Bertozzi C. R. (2010). Chem. Soc. Rev..

[cit5] Baskin J. M., Prescher J. A., Laughlin S. T., Agard N. J., Chang P. V., Miller I. A., Lo A., Codelli J. A., Bertozzi C. R. (2007). Proc. Natl. Acad. Sci. U.S.A..

[cit6] Blackman M. L., Royzen M., Fox J. M. (2008). J. Am. Chem. Soc..

[cit7] Debets M. F., van Hest J. C., Rutjes F. P. (2013). Org. Biomol. Chem..

[cit8] Earl J. C., Mackney A. W. (1935). J. Chem. Soc..

[cit9] Huisgen R., Grashey R., Gotthardt H., Schmidt R. (1962). Angew. Chem., Int. Ed. Engl..

[cit10] Browne D. L., Harrity J. P. (2010). Tetrahedron.

[cit11] Kolodych S., Rasolofonjatovo E., Chaumontet M., Nevers M. C., Créminon C., Taran F. (2013). Angew. Chem., Int. Ed..

[cit12] Wallace S., Chin J. W. (2014). Chem. Sci..

[cit13] Shortly after Chin's report (ref. 12), 4-chloro-sydnones were reported to react much faster with BCN than non-chlorinated analogues: Plougastel L. Koniev O. Specklin S. Decuypere E. Créminon C. Buisson D.-A. Wagner A. Kolodych S. Taran F., Chem. Commun., 2014, 50 , 9376 –9378 , . For example, the rate constant for the (3 + 2) cycloaddition between *N*-phenyl 4-chloro-sydnone and BCN in PBS/DMSO was reported to be 0.872 M^–1^ s^–1^ at 25 °C . 10.1039/c4cc03816a 25005038

[cit14] Frisch M. J., Trucks G. W., Schlegel H. B., Scuseria G. E., Robb M. A., Cheeseman J. R., Scalmani G., Barone V., Mennucci B., Petersson G. A., Nakatsuji H., Caricato M., Li X., Hratchian H. P., Izmaylov A. F., Bloino J., Zheng G., Sonnenberg J. L., Hada M., Ehara M., Toyota K., Fukuda R., Hasegawa J., Ishida M., Nakajima T., Honda Y., Kitao O., Nakai H., Vreven T., Montgomery Jr. J. A., Peralta J. E., Ogliaro F., Bearpark M., Heyd J. J., Brothers E., Kudin K. N., Staroverov V. N., Kobayashi R., Normand J., Raghavachari K., Rendell A., Burant J. C., Iyengar S. S., Tomasi J., Cossi M., Rega N., Millam J. M., Klene M., Knox J. E., Cross J. B., Bakken V., Adamo C., Jaramillo J., Gomperts R., Stratmann R. E., Yazyev O., Austin A. J., Cammi R., Pomelli C., Ochterski J. W., Martin R. L., Morokuma K., Zakrzewski V. G., Voth G. A., Salvador P., Dannenberg J. J., Dapprich S., Daniels A. D., Farkas Ö., Foresman J. B., Ortiz J. V., Cioslowski J. and Fox D. J., Gaussian 09, Revision D.01, Gaussian Inc., Wallingford, CT, 2013.

[cit15] Zhao Y., Truhlar D. G. (2008). Theor. Chem. Acc..

[cit16] Liang Y., Mackey J. L., Lopez S. A., Liu F., Houk K. N. (2012). J. Am. Chem. Soc..

[cit17] Gordon C. G., Mackey J. L., Jewett J. C., Sletten E. M., Houk K. N., Bertozzi C. R. (2012). J. Am. Chem. Soc..

[cit18] Karver M. R., Weissleder R., Hilderbrand S. A. (2011). Bioconjugate Chem..

[cit19] Sanders B. C., Friscourt F., Ledin P. A., Mbua N. E., Arumugam S., Guo J., Boltje T. J., Popik V. V., Boons G.-J. (2010). J. Am. Chem. Soc..

[cit20] Karver M. R., Weissleder R., Hilderbrand S. A. (2012). Angew. Chem., Int. Ed..

