# Supplementary material for: Discovery of new mutually orthogonal bioorthogonal cycloaddition pairs through computational screening
Source: Chem Sci. 2015 Nov 11;7(2):1257–61. doi: 10.1039/C5SC03259H (PMC4763938; doi:10.1039/C5SC03259H)
Supplement: Supplementary file 1 [file SC-007-C5SC03259H-s001.pdf]

Electronic Supplementary Material (ESI) for Chemical Science.
This journal is © The Royal Society of Chemistry 2015

Supplementary Information

Discovery of new mutually orthogonal bioorthogonal cycloaddition pairs through computational screening

Maruthi Kumar Narayanam,[a] Yong Liang,[b] K. N. Houk*,[b,c] and Jennifer M. Murphy*,[a]

[a]Crump Institute for Molecular Imaging, David Geffen School of Medicine, [b]Department of Chemistry and Biochemistry, and [c]Department of Chemical and Biomolecular Engineering, University of California, Los Angeles, California 90095, United States

Table of Contents

Computational Details……………………………………………………………………...S3

Coordinates and Energies of Computed Stationary Points…………………………………S3-S14

Materials and Methods………………………………………………………………….S15-S17

Synthesis of Sydnone and Tetrazine Compounds…………………………………………S18-S22

Figure S1. Analytical HPLC trace of purified BARAC………………………………..S23

Synthesis of Fluorophore Conjugates…………………………………………….......S24-S25

Figure S2. Analytical HPLC trace of purified Syd-630………………………………..S24

Figure S3. Analytical HPLC trace of purified Tz-504……………………………….....S25

Reaction Rate Determination……………………………………………….……..S26-S36

Figure S4. Rate Determination of BARAC and DIBAC with benzyl azide...………....S26

Figure S5. Rate Determination of diaryltetrazine with norbornene acetic acid………..S27

Figure S6. Phenyl sydnone and norbornene acetic acid cross reactivity.....……………S29

Figure S7. Diaryltetrazine and DIBAC cross reactivity……………….………………S30

Figure S8. Phenyl sydnone and BARAC cycloadduct…………………………………S31

Figure S9. Phenyl sydnone and DIBAC cycloadduct………..…………………………S32

Figure S10. Benzyl azide and DIBAC cycloadduct……………………….……….....…S33

Figure S11. Benzyl azide and BARAC cycloadduct………………....………………....S34

Figure S12. Competition experiment with BARAC.…….…………………………..…S35

Figure S13. Competition experiment with DIBAC……...……………………………..S36

Figure S14. The ^{13}C NMR spectra of cycloadducts **7** and **11**...………………………...S37

References……………………………………………………………………………....S38

Computational Details

Calculations were performed with the Gaussian 09.[1] The geometry optimization of all the minima and transition states involved was carried out at the M06-2X level of theory[2] with the 6-31G(d) basis set.[3] The vibrational frequencies were computed at the same level to check whether each optimized structure is an energy minimum or a transition state and to evaluate its zero-point vibration energy (ZPVE) and thermal corrections at 298 K. A quasiharmonic correction was applied during the entropy calculation by setting all positive frequencies that are less than 100 cm^{-1} to 100 cm^{-1}.[4] The single-point energies and solvent effects in water were computed at the M06-2X/6-311+G(d,p) level using the gas-phase optimized structures at the M06-2X/6-31G(d) level. Solvation energies were evaluated by a self-consistent reaction field (SCRF) using the CPCM model,[5] where UFF radii were used.

Coordinates and Energies of Computed Stationary Points

1

G(water) = -568.166377 Hartree

N	-0.635202	-0.097848	0.044462
N	-1.176785	-1.187722	0.493203
C	-1.502844	0.851138	-0.344817
H	-1.199579	1.781075	-0.789803
C	-2.800581	0.306340	-0.135349
O	-2.499658	-0.979723	0.391500
O	-3.933755	0.670306	-0.300094
C	0.796660	-0.016002	0.014204
C	1.406241	1.202564	0.287645
C	1.528477	-1.158553	-0.289277
C	2.794428	1.276933	0.246043
H	0.806374	2.067625	0.550280
C	2.915531	-1.068460	-0.318160
H	1.010921	-2.087698	-0.498790
C	3.547865	0.145056	-0.055933
H	3.286037	2.220183	0.459064
H	3.502101	-1.949492	-0.555884
H	4.630699	0.208534	-0.085905

4

G(water) = -464.354502 Hartree

C	-2.418782	0.603908	-0.474622
C	-2.419220	-0.604006	-0.474713
C	-1.853064	1.930277	-0.212649
H	-1.585646	2.436690	-1.147594
H	-2.545200	2.586350	0.325644
C	-1.853173	-1.930263	-0.212568
H	-1.585673	-2.436694	-1.147482
H	-2.545191	-2.586460	0.325733
C	-0.585477	-1.666162	0.639194
H	-0.902363	-1.227523	1.592819
H	-0.099332	-2.622607	0.872795
C	-0.585294	1.666398	0.639113
H	-0.902107	1.227905	1.592828
H	-0.099172	2.622899	0.872515
C	0.421196	0.760342	-0.046069
C	0.421077	-0.760220	-0.046041
C	1.449436	0.000000	0.742622
H	1.301730	0.000031	1.822755
H	0.801371	-1.166710	-0.982214
H	0.801528	1.166755	-0.982258
C	2.902583	-0.000124	0.334074

H	3.401737	0.888842	0.750093
H	3.401574	-0.889186	0.750083
O	2.972285	-0.000122	-1.079750
H	3.902774	-0.000215	-1.337767

TS1
G(water) = -1032.485500 Hartree

N	2.450207	-0.583557	-0.026148
N	2.075666	-1.035230	1.169697
C	2.035024	-1.413617	-0.997222
H	2.252441	-1.248328	-2.040800
C	1.860969	-2.702017	-0.350928
O	1.979097	-2.399743	0.997748
O	1.657962	-3.812066	-0.762704
C	2.667862	0.816676	-0.135902
C	3.451893	1.443097	0.829379
C	2.089692	1.530377	-1.181085
C	3.666106	2.812548	0.734973
H	3.879061	0.851497	1.631740
C	2.324718	2.898986	-1.269875
H	1.445288	1.024890	-1.892775
C	3.110419	3.540317	-0.316090
H	4.276332	3.311410	1.480817
H	1.877523	3.465811	-2.079976
H	3.286446	4.608737	-0.388738
C	-0.064668	-0.722876	0.738250
C	-0.152258	-1.046527	-0.454962
C	-0.670385	-0.319750	2.016495
H	-0.290074	-0.947511	2.830268
H	-0.383179	0.712394	2.255287
C	-0.996593	-1.370279	-1.623039
H	-0.723419	-2.353500	-2.025617
H	-0.833455	-0.640577	-2.428204
C	-2.476282	-1.358315	-1.194996
H	-3.101744	-1.619439	-2.058486
H	-2.624863	-2.145950	-0.447239
C	-2.922199	-0.018416	-0.646485
C	-2.202177	-0.441412	1.906721
H	-2.654924	-0.149496	2.863262
H	-2.456321	-1.496165	1.747324
C	-2.790431	0.411988	0.802166
H	-2.798993	0.807674	-1.345375
H	-2.589972	1.476765	0.911142
C	-4.140473	0.097732	0.223984
H	-4.579668	-0.846312	0.546609
C	-5.158052	1.187643	-0.009171
H	-5.879998	0.859496	-0.772958
H	-5.716602	1.375201	0.920887
O	-4.478365	2.356634	-0.430325
H	-5.133515	3.049389	-0.583044

8
G(water) = -1032.556846 Hartree

N	-1.597911	-0.439798	-0.116976
N	-1.067747	0.006276	1.094441
C	-1.100431	0.669448	-0.937212
H	-1.365053	0.674519	-1.992315
C	-1.638959	1.865013	-0.115413
O	-1.629641	1.452304	1.168165
O	-1.968698	2.953194	-0.494609
C	-2.995859	-0.723685	-0.106421
C	-3.746611	-0.703669	1.068261
C	-3.593833	-1.074098	-1.318113
C	-5.096748	-1.038843	1.018687
H	-3.273504	-0.421384	2.001031
C	-4.945816	-1.391111	-1.355883
H	-2.993626	-1.111991	-2.222638
C	-5.703512	-1.377672	-0.186756
H	-5.679777	-1.022989	1.934350
H	-5.405600	-1.659283	-2.302063
H	-6.758497	-1.630073	-0.216318
C	0.351161	0.255355	0.730756
C	0.376586	0.655311	-0.549942
C	1.247015	0.066203	1.911727
H	0.821735	0.683549	2.715411
H	1.116067	-0.972652	2.244941
C	1.363687	1.130475	-1.573249
H	0.993908	2.096063	-1.946235
H	1.289400	0.442185	-2.428618
C	2.831922	1.267791	-1.180189
H	3.375581	1.648778	-2.053450
H	2.938917	2.031051	-0.402340
C	3.447148	-0.039100	-0.741692
C	2.733259	0.368140	1.755261
H	3.213735	0.188094	2.724427
H	2.876363	1.432681	1.542130
C	3.400123	-0.481984	0.700660
H	3.367875	-0.847601	-1.465797

H	3.291217	-1.555669	0.840345
C	4.699872	-0.053110	0.085449
H	5.079017	0.919796	0.397669
C	5.784041	-1.066777	-0.189357
H	6.449099	-0.688981	-0.981043
H	6.390778	-1.210611	0.717862
O	5.171826	-2.280878	-0.582695
H	5.865808	-2.923922	-0.775937

TS2

G(water) = -1032.555044 Hartree

N	-1.613544	-0.446329	-0.123876
N	-1.057255	-0.094975	1.072765
C	-1.094519	0.658891	-0.925598
H	-1.369159	0.693336	-1.977869
C	-1.615964	1.866490	-0.070016
O	-1.621104	1.468733	1.191918
O	-1.908794	2.953855	-0.496286
C	-3.013919	-0.720525	-0.115950
C	-3.766972	-0.670884	1.055566
C	-3.607802	-1.076837	-1.326857
C	-5.121932	-0.985118	1.003053
H	-3.292271	-0.379073	1.984539
C	-4.964674	-1.372347	-1.367574
H	-3.001795	-1.133725	-2.226500
C	-5.726575	-1.330859	-0.201793
H	-5.710308	-0.947139	1.914544
H	-5.424851	-1.645748	-2.311950
H	-6.785284	-1.566999	-0.233467
C	0.345577	0.180960	0.725642
C	0.373462	0.624671	-0.544305
C	1.238504	-0.016204	1.909295
H	0.798181	0.582029	2.719222
H	1.124261	-1.062309	2.224753
C	1.359591	1.152452	-1.542591
H	0.974938	2.125333	-1.879862
H	1.300673	0.496260	-2.423877
C	2.823149	1.297613	-1.136376
H	3.366158	1.714468	-1.993484
H	2.914650	2.036607	-0.333655
C	3.453117	-0.014186	-0.735831
C	2.720620	0.311792	1.767220
H	3.199749	0.112878	2.733367
H	2.848843	1.383555	1.583672
C	3.403904	-0.500027	0.692939
H	3.387106	-0.801320	-1.484415
H	3.307563	-1.578577	0.801337
C	4.701354	-0.037865	0.097619
H	5.067677	0.929862	0.439740
C	5.798763	-1.030293	-0.201298
H	6.467416	-0.618743	-0.972839
H	6.398160	-1.199272	0.706398
O	5.202986	-2.236230	-0.642193
H	5.905093	-2.869514	-0.838250

5

G(water) = -844.111532 Hartree

N	-2.043200	0.147851	-0.228940
N	-1.296930	-0.938846	-0.504146
C	-1.284198	1.277645	-0.199857
H	-1.705268	2.245132	0.031027
C	-3.433110	0.019317	-0.003100
C	-3.970085	-1.243638	0.250481
C	-4.261162	1.141896	-0.034886
C	-5.334432	-1.372001	0.479310
H	-3.307987	-2.100652	0.259171
C	-5.623162	0.998784	0.207834
H	-3.854177	2.120205	-0.266451
C	-6.167690	-0.255406	0.465992
H	-5.748591	-2.356521	0.674289
H	-6.260995	1.876829	0.182974
H	-7.231710	-0.362942	0.649696
C	-0.057922	-0.496380	-0.658827
C	0.011938	0.916164	-0.476049
C	1.034482	-1.477775	-0.958826
H	0.548441	-2.445628	-1.109769
H	1.526705	-1.214174	-1.903912
C	1.194624	1.838097	-0.525190
H	0.821347	2.862886	-0.422650
H	1.673770	1.784604	-1.511624
C	2.261563	1.562291	0.559721
H	2.663107	2.515199	0.925243
H	1.787198	1.080132	1.422469
C	3.417885	0.731550	0.049440
C	2.104968	-1.580848	0.151741
H	2.406401	-2.628845	0.266491
H	1.666709	-1.284030	1.112058
C	3.343440	-0.764699	-0.145375

H	4.026716	1.235332	-0.699122
H	3.908985	-1.116607	-1.006416
C	4.188156	-0.187531	0.953502
H	3.792120	-0.300030	1.962296
C	5.694419	-0.252701	0.884086
H	6.126911	0.536628	1.518354
H	6.039756	-1.222211	1.274944
O	6.091077	-0.085391	-0.465134
H	7.054557	-0.133812	-0.506374

CO_2
G(water) = -188.586344 Hartree

C	0.000000	0.000000	0.000000
O	0.000000	0.000000	1.162797
O	0.000000	0.000000	-1.162797

----

norbornene
G(water) = -272.545754 Hartree

C	-1.275344	-0.668267	-0.500391
C	-0.084771	-1.124602	0.325215
C	1.179937	-0.777633	-0.521120
C	1.179960	0.777613	-0.521143
C	-1.275321	0.668278	-0.500390
C	-0.084742	1.124606	0.325239
C	-0.030779	0.000005	1.376394
H	-1.915633	-1.325220	-1.079425
H	-0.114261	-2.151779	0.692510
H	2.080314	-1.172541	-0.039936
H	1.123410	-1.201886	-1.526509
H	1.123255	1.201826	-1.526539
H	2.080396	1.172565	-0.040118
H	-1.915564	1.325267	-1.079429
H	-0.114276	2.151779	0.692547
H	-0.897291	0.000013	2.042551
H	0.896013	-0.000019	1.961528

TS3
G(water) = -840.664333 Hartree

N	0.891370	1.070349	-0.090828
N	0.181916	0.938467	-1.222891
C	0.083895	1.464115	0.919097
H	0.447935	1.668631	1.913101
C	-1.011054	2.158065	0.278356
O	-0.854231	1.830055	-1.061863
O	-1.913431	2.844682	0.676276
C	2.096352	0.328944	0.005758
C	2.834792	0.102185	-1.156059
C	2.528327	-0.157582	1.238232
C	4.017567	-0.621447	-1.073606
H	2.473781	0.494896	-2.099519
C	3.722829	-0.868404	1.305088
H	1.939972	0.006648	2.134676
C	4.468208	-1.104095	0.153941
H	4.594235	-0.802335	-1.974955
H	4.064054	-1.246175	2.263379
H	5.396407	-1.662983	0.211921
C	-0.820723	-0.829432	-0.537586
C	-2.178058	-1.037616	-1.185189
C	-2.579841	-0.528351	0.975510
C	-1.067204	-0.517718	0.801837
H	0.036700	-1.411791	-0.864266
H	-0.426646	-0.864018	1.609225
C	-3.077257	-0.064192	-0.404102
H	-4.138700	-0.294519	-0.545845
H	-2.919070	0.988935	-0.620821
C	-2.679757	-2.391731	-0.593427
H	-3.595195	-2.707807	-1.103589
H	-1.940925	-3.189722	-0.708938
C	-2.960847	-2.039171	0.898277
H	-4.019026	-2.174832	1.143657
H	-2.376224	-2.647722	1.594178
H	-2.201412	-0.978835	-2.274675
H	-2.970963	0.000604	1.846434

Cp(3,3)
G(water) = -195.125864 Hartree

C	1.262218	0.000000	-0.647393
C	1.262251	0.000000	0.647586
C	-0.094543	0.000000	-0.000408
H	1.808131	0.000000	1.579301
C	-0.937031	-1.267285	0.000024
H	-1.586720	-1.305168	0.882845
H	-1.585151	-1.306582	-0.883882
H	-0.304718	-2.159209	0.001161

C	-0.937031	1.267285	0.000024
H	-1.585151	1.306582	-0.883882
H	-1.586720	1.305169	0.882845
H	-0.304717	2.159209	0.001161
H	1.809858	0.000000	-1.578546

TS4

G(water) = -763.244603 Hartree

N	-0.473948	-0.958700	-0.028822
N	0.223306	-0.817835	-1.157539
C	0.340916	-1.226608	1.008421
H	-0.022515	-1.412285	2.006197
C	1.529774	-1.805649	0.419434
O	1.363829	-1.546037	-0.936711
O	2.486190	-2.384484	0.854270
C	1.212348	0.918479	0.771892
C	0.938825	1.119458	-0.536502
C	-1.768163	-0.370371	0.010683
C	-2.549732	-0.409214	-1.142694
C	-2.229996	0.227672	1.180000
C	-3.814406	0.165492	-1.116702
H	-2.158234	-0.885070	-2.034510
C	-3.504100	0.785893	1.193333
H	-1.597798	0.274227	2.060324
C	-4.296400	0.758363	0.048655
H	-4.429889	0.140552	-2.009908
H	-3.872154	1.254048	2.100365
H	-5.287655	1.199422	0.064230
C	2.350387	1.388779	-0.098262
H	0.819566	1.243026	1.727253
H	0.210063	1.643746	-1.141235
C	2.747860	2.857295	0.031016
H	3.173689	3.224649	-0.910133
H	1.889059	3.484966	0.287739
H	3.510655	2.982648	0.808408
C	3.554463	0.539648	-0.479667
H	3.942248	0.872495	-1.449445
H	4.348578	0.688858	0.260856
H	3.358067	-0.526788	-0.543731

----

cyclooctyne

G(water) = -311.786954 Hartree

C	0.681129	1.341424	0.376746
C	-0.682343	1.341190	-0.376880
C	0.603634	-1.453056	0.028187
C	-1.857400	0.581825	0.279100
C	-0.602258	-1.452377	-0.028549
C	-1.957951	-0.910097	-0.119865
C	1.856814	0.582765	-0.278986
C	1.958707	-0.909006	0.120049
H	-0.539204	0.960586	-1.396591
H	-1.762279	0.646840	1.369540
H	-0.998167	2.383992	-0.489899
H	-2.803074	1.070506	0.016803
H	-2.672084	-1.434049	0.523759
H	-2.326611	-0.999077	-1.149093
H	2.802035	1.072273	-0.016545
H	1.761937	0.647589	-1.369447
H	2.673865	-1.432067	-0.523167
H	2.326957	-0.997634	1.149457
H	0.996525	2.384355	0.489920
H	0.538104	0.960678	1.396452

TS5

G(water) = -879.914408 Hartree

N	1.256500	1.180328	-0.154197
N	0.657433	1.110070	-1.347490
C	0.437732	1.773290	0.733549
H	0.719124	1.952625	1.760147
C	-0.438582	2.617205	-0.063162
O	-0.187592	2.203085	-1.360937
O	-1.230247	3.475843	0.217333
C	-1.136387	0.217061	0.497435
C	-0.879839	-0.237344	-0.631024
C	-2.015248	0.306935	1.682330
H	-1.556274	-0.232990	2.522628
H	-2.121048	1.352864	1.996089
C	-1.105880	-1.133984	-1.773741
H	-0.152388	-1.519956	-2.151480
H	-1.543944	-0.544906	-2.590808
C	-3.400219	-0.281740	1.376460
H	-3.981261	-0.266653	2.306056
H	-3.914541	0.386908	0.675858
C	-3.385694	-1.711790	0.803964
H	-2.540438	-2.265528	1.235675
H	-4.286403	-2.215304	1.171055

C	-2.040977	-2.290032	-1.390819
H	-2.252355	-2.863022	-2.301346
H	-1.508806	-2.968618	-0.712473
C	-3.368339	-1.859944	-0.740177
H	-4.116906	-2.613862	-1.006802
H	-3.711486	-0.924726	-1.203015
C	2.238019	0.189609	0.117320
C	3.226759	-0.034494	-0.837885
C	2.193436	-0.537453	1.303078
C	4.194080	-1.000447	-0.591167
H	3.222972	0.550418	-1.751028
C	3.178060	-1.492083	1.542456
H	1.387214	-0.374373	2.010771
C	4.176170	-1.724549	0.600062
H	4.970239	-1.180720	-1.327698
H	3.154559	-2.064741	2.463889
H	4.938378	-2.473065	0.791053

Cp(1,3)
G(water) = -195.131186 Hartree

C	-0.685716	0.206784	0.034439
C	0.003444	1.281046	-0.188019
C	0.743755	0.170246	0.498557
H	0.071276	2.282536	-0.585351
C	-1.930745	-0.588044	-0.047853
H	-2.268156	-0.869273	0.955190
H	-2.729430	-0.032687	-0.545229
H	-1.751504	-1.516576	-0.599893
C	1.793558	-0.656829	-0.223730
H	2.785056	-0.196606	-0.147229
H	1.865357	-1.666757	0.195570
H	1.544061	-0.746137	-1.286163
H	0.937560	0.266279	1.572748

TS6
G(water) = -763.259364 Hartree

N	-0.257098	-1.037201	-0.147597
N	0.414872	-0.632876	-1.227803
C	0.585388	-1.447593	0.817347
H	0.245168	-1.846384	1.759436
C	1.811314	-1.803478	0.135457
O	1.608782	-1.300756	-1.148109
O	2.820725	-2.365849	0.456680
C	1.422442	0.724063	0.972705
C	1.104560	1.219499	-0.239994
C	-1.571675	-0.525197	0.019100
C	-2.402154	-0.448244	-1.097192
C	-1.995250	-0.089021	1.271321
C	-3.679212	0.077621	-0.948631
H	-2.034592	-0.792832	-2.057210
C	-3.282850	0.421738	1.406582
H	-1.320614	-0.126033	2.120074
C	-4.123801	0.508553	0.300822
H	-4.333195	0.144797	-1.811898
H	-3.622239	0.764738	2.378427
H	-5.124510	0.913506	0.410793
C	2.561384	1.081775	0.067039
H	1.175392	0.869823	2.015621
C	3.415168	2.308978	0.334535
H	3.838766	2.704715	-0.594600
H	2.825232	3.103073	0.805145
H	4.247487	2.065360	1.002603
H	3.108818	0.264397	-0.411412
C	0.226280	2.219547	-0.909280
H	0.187613	2.055131	-1.988997
H	-0.793633	2.189078	-0.512886
H	0.630276	3.224557	-0.734734

DIFO
G(water) = -510.299493 Hartree

C	-0.329082	1.579977	0.266345
C	-1.673885	1.181350	-0.409347
C	0.409933	-1.178338	-0.075735
C	-2.539058	0.122782	0.310012
C	-0.764313	-1.456068	-0.066028
C	-2.218621	-1.340393	-0.083054
C	0.972801	1.162080	-0.444633
C	1.505066	-0.202185	-0.016281
H	-1.487460	0.851540	-1.439914
H	-2.413455	0.229989	1.393485
H	-2.282352	2.087562	-0.493422
H	-3.598064	0.303551	0.095483
H	-2.704303	-2.044370	0.599352
H	-2.590949	-1.550943	-1.092420
H	1.775989	1.877226	-0.234915
H	0.833271	1.130813	-1.530264

H	-0.307370	2.670707	0.350928
H	-0.296885	1.202829	1.295757
F	2.563487	-0.544981	-0.797018
F	1.980350	-0.118811	1.260158

TS7

G(water) = -1078.430546 Hartree

N	-1.424517	1.217157	0.110567
N	-1.081531	1.406460	1.376876
C	-0.527031	1.755730	-0.729108
H	-0.639005	1.732950	-1.801886
C	0.150159	2.783126	0.049904
O	-0.275787	2.518989	1.347930
O	0.893124	3.681760	-0.220672
C	-2.302509	0.127372	-0.160919
C	-3.473646	0.027646	0.584311
C	-1.967694	-0.804888	-1.138668
C	-4.338193	-1.030611	0.333109
H	-3.688528	0.775956	1.339468
C	-2.850666	-1.853082	-1.383270
H	-1.022489	-0.729836	-1.667416
C	-4.031429	-1.966245	-0.653671
H	-5.257683	-1.118624	0.902438
H	-2.605978	-2.589540	-2.141771
H	-4.713350	-2.787049	-0.851735
C	0.853709	-0.803736	2.369446
C	2.103751	-1.690764	2.186755
C	2.082289	-2.480126	0.856832
C	3.133182	-2.085129	-0.198996
C	3.244325	-0.619150	-0.649083
C	1.948082	0.022028	-1.117972
C	1.024571	0.284277	-0.003773
C	0.692080	-0.000235	1.151267
F	2.225023	1.172565	-1.792794
F	1.333742	-0.803729	-2.033637
H	0.939244	-0.160848	3.251831
H	-0.041759	-1.421466	2.513937
H	2.175236	-2.376008	3.038061
H	2.991151	-1.047615	2.230009
H	2.242197	-3.543632	1.064338
H	1.084755	-2.409696	0.407320
H	2.953152	-2.699000	-1.088514
H	4.124903	-2.369841	0.173029
H	3.638583	0.017941	0.148580
H	3.948457	-0.561808	-1.486947

TCO

G(water) = -312.998444 Hartree

C	-0.413966	-0.522065	-1.358397
H	-1.490184	-0.336794	-1.334889
C	0.413966	0.522065	-1.358397
H	1.490184	0.336794	-1.334889
C	0.034981	-1.871510	-0.901221
H	-0.510280	-2.704358	-1.358222
H	1.102540	-2.008137	-1.112700
C	-0.034981	1.871510	-0.901221
H	-1.102540	2.008137	-1.112700
H	0.510280	2.704358	-1.358222
C	0.183001	1.877834	0.635533
H	1.260794	1.924503	0.841500
H	-0.248046	2.798155	1.048278
C	-0.413966	0.660993	1.376189
H	-0.560669	0.965017	2.418884
H	-1.420918	0.465721	0.984525
C	-0.183001	-1.877834	0.635533
H	0.248046	-2.798155	1.048278
H	-1.260794	-1.924503	0.841500
C	0.413966	-0.660993	1.376189
H	0.560669	-0.965017	2.418884
H	1.420918	-0.465721	0.984525

TS8

G(water) = -881.130103 Hartree

N	1.116510	1.288786	-0.143758
N	0.530572	1.203850	-1.343935
C	0.285330	1.868033	0.744726
H	0.562478	2.063356	1.768374
C	-0.621095	2.671779	-0.047219
O	-0.371130	2.247862	-1.345062
O	-1.444816	3.504480	0.220861
C	2.161779	0.367727	0.127987
C	3.039804	0.035012	-0.902718
C	2.291746	-0.192439	1.396507
C	4.054571	-0.879593	-0.654097
H	2.910768	0.490714	-1.877838
C	3.322294	-1.098043	1.632703

H	1.589016	0.058075	2.183961
C	4.200965	-1.446309	0.611535
H	4.739586	-1.145352	-1.452637
H	3.428285	-1.539193	2.618421
H	4.998236	-2.157769	0.799971
C	-0.954926	-0.250470	-0.777668
H	-1.628665	0.110793	-1.555006
C	-1.242191	0.133503	0.522664
H	-0.742129	-0.427537	1.316355
C	-0.328435	-1.585589	-1.055714
H	0.253740	-1.581621	-1.984155
H	0.354116	-1.857919	-0.239344
C	-2.606254	0.652918	0.879223
H	-3.008951	1.235548	0.041168
H	-2.589747	1.321484	1.747717
C	-3.531984	-0.547771	1.164536
H	-3.226639	-1.021358	2.107832
H	-4.547136	-0.166774	1.328774
C	-3.569077	-1.621428	0.059147
H	-4.516216	-2.161341	0.167115
H	-3.626585	-1.126310	-0.919429
C	-1.452993	-2.639923	-1.148699
H	-0.991010	-3.627884	-1.263450
H	-2.031234	-2.463289	-2.065553
C	-2.419284	-2.669616	0.051760
H	-2.872249	-3.667046	0.075947
H	-1.839176	-2.590512	0.981199

DIBAC

G(water) = -785.235091 Hartree

C	1.792874	-1.272296	0.126711
C	1.745302	0.101539	-0.228318
C	2.934150	0.759350	-0.538324
C	4.154740	0.094635	-0.457710
C	4.199516	-1.249526	-0.094739
C	3.022046	-1.932408	0.185954
H	2.893238	1.799067	-0.849368
H	5.070937	0.626362	-0.693658
H	5.151013	-1.768365	-0.039702
H	3.040084	-2.982878	0.456690
C	0.511670	-1.854456	0.359067
C	-0.700455	-1.811226	0.393815
C	-1.973158	-1.207373	0.158655
C	-3.202887	-1.651505	0.647399
C	-1.881186	-0.014442	-0.597546
C	-4.349338	-0.905905	0.399387
H	-3.247866	-2.569310	1.224231
C	-3.038413	0.726491	-0.809560
C	-4.265882	0.284912	-0.319439
H	-5.307407	-1.247608	0.778171
H	-2.967716	1.665686	-1.350525
H	-5.159573	0.875285	-0.496177
C	-0.559765	0.402912	-1.220638
N	0.502604	0.794068	-0.268845
H	-0.159080	-0.422280	-1.814735
H	-0.741841	1.253657	-1.883558
C	0.261942	1.962841	0.427708
O	-0.731419	2.626376	0.188166
C	1.239576	2.360256	1.516372
H	1.700126	1.498114	2.002256
H	2.035686	2.988962	1.107396
H	0.681126	2.951026	2.242812

TS9

G(water) = -1353.368534 Hartree

N	2.508173	-0.788848	0.803351
N	2.167365	0.173223	1.659314
C	1.798220	-1.902491	1.045374
H	1.927169	-2.811222	0.477472
C	1.409290	-1.811967	2.443352
O	1.718444	-0.501155	2.771100
O	0.935811	-2.593861	3.220880
C	3.018255	-0.364139	-0.456325
C	4.030618	0.590535	-0.473404
C	2.478814	-0.883234	-1.630276
C	4.519356	1.025766	-1.699353
H	4.419769	0.972218	0.464284
C	2.988842	-0.446493	-2.849200
H	1.658329	-1.593602	-1.588207
C	4.004832	0.505051	-2.885534
H	5.310317	1.768052	-1.728055
H	2.579210	-0.843184	-3.772286
H	4.393919	0.845510	-3.839614
C	-0.130699	1.757895	0.607752
C	-1.344988	2.092485	-0.038740
C	-1.665308	3.433813	-0.245022
C	-0.829920	4.443769	0.220415
C	0.358320	4.118094	0.869354

C	0.709784	2.786339	1.049059
H	-2.577041	3.676699	-0.782580
H	-1.101394	5.482620	0.061325
H	1.017258	4.902173	1.228336
H	1.637590	2.517503	1.540078
C	0.183213	0.361538	0.743554
C	-0.116711	-0.832187	0.531523
C	-1.018238	-1.795980	-0.050275
C	-1.080857	-3.132424	0.361900
C	-1.877991	-1.327982	-1.066050
C	-1.990386	-3.999416	-0.230492
H	-0.442413	-3.470964	1.174095
C	-2.786893	-2.210785	-1.641368
C	-2.841427	-3.541078	-1.234554
H	-2.043378	-5.031918	0.100555
H	-3.473908	-1.835867	-2.394230
H	-3.559636	-4.216430	-1.688874
C	-1.792885	0.112583	-1.518325
N	-2.226503	1.074427	-0.487378
H	-0.760541	0.368280	-1.774037
H	-2.420332	0.252404	-2.403833
C	-3.575255	1.051065	-0.183572
O	-4.334295	0.331338	-0.808034
C	-4.058766	1.901471	0.974791
H	-3.287148	2.064413	1.729008
H	-4.404365	2.875567	0.617254
H	-4.911665	1.382200	1.413228

BARAC
G(water) = -745.938498 Hartree

C	-1.874345	0.946253	-0.046579
C	-1.796379	-0.456209	0.179056
C	-2.953623	-1.218932	0.187671
C	-4.191524	-0.610544	-0.017605
C	-4.272192	0.762805	-0.233035
C	-3.119108	1.541793	-0.251859
H	-2.870049	-2.288528	0.351415
H	-5.094338	-1.212808	-0.011905
H	-5.238292	1.230581	-0.393245
H	-3.174474	2.610524	-0.429771
C	-0.598175	1.595360	-0.070843
C	0.616293	1.598252	-0.025277
C	1.894362	0.944715	-0.024578
C	3.143192	1.537424	0.162247
C	1.802680	-0.453755	-0.238025
C	4.289678	0.748303	0.138868
H	3.209023	2.608811	0.320014
C	2.956224	-1.223828	-0.283935
C	4.199229	-0.624145	-0.082749
H	5.260952	1.209454	0.287083
H	2.881541	-2.288865	-0.482749
H	5.100148	-1.228738	-0.109988
C	0.422195	-0.987705	-0.564580
N	-0.515442	-1.031790	0.470939
C	-0.097584	-0.888656	1.862493
H	-0.035788	0.161536	2.174681
H	-0.821718	-1.407155	2.495872
H	0.878208	-1.358870	1.996367
O	0.123418	-1.260024	-1.705247

TS10
G(water) = -1314.074462 Hartree

N	-1.583462	-1.725378	-0.831647
N	-1.753715	-0.733992	-1.704238
C	-0.423464	-2.358736	-1.060724
H	-0.099330	-3.209292	-0.481382
C	-0.116534	-2.105851	-2.459046
O	-1.028066	-1.119579	-2.806309
O	0.685035	-2.567132	-3.222430
C	-2.245462	-1.591719	0.420971
C	-3.573409	-1.174593	0.431020
C	-1.551327	-1.851874	1.599403
C	-4.217870	-1.019972	1.652561
H	-4.078747	-0.981321	-0.509023
C	-2.215414	-1.705362	2.813550
H	-0.502083	-2.130709	1.568225
C	-3.543904	-1.289342	2.842551
H	-5.252841	-0.695142	1.673772
H	-1.684520	-1.904051	3.738817
H	-4.053394	-1.171466	3.793342
C	1.990830	-0.822826	0.010735
C	2.564235	0.093596	0.926549
C	3.771086	-0.194111	1.545594
C	4.434958	-1.384775	1.257410
C	3.893331	-2.280183	0.337730
C	2.679339	-2.004916	-0.282678
H	4.184887	0.531476	2.238501
H	5.382425	-1.605094	1.738847

H	4.423354	-3.195743	0.095058
H	2.276985	-2.683733	-1.030288
C	0.722732	-0.455910	-0.577007
C	-0.133526	0.427102	-0.803471
C	-0.557300	1.806050	-0.707449
C	-1.813748	2.272314	-1.104789
C	0.377804	2.724797	-0.175244
C	-2.131185	3.620440	-0.973684
H	-2.526052	1.568745	-1.519923
C	0.061714	4.072745	-0.074796
C	-1.199365	4.520975	-0.462306
H	-3.110029	3.972328	-1.284231
H	0.806201	4.767972	0.301754
H	-1.448977	5.573894	-0.376509
C	1.748492	2.183021	0.132534
N	1.846047	1.297643	1.208150
C	0.775651	1.176109	2.190828
H	-0.005520	0.469068	1.882406
H	1.211524	0.838534	3.135029
H	0.320412	2.155189	2.350438
O	2.693983	2.422907	-0.584027

9

G(water) = -790.263948 Hartree

C	1.255694	-0.000009	-0.000038
C	-1.301857	-0.000012	-0.000010
N	-0.683032	-1.172266	0.185884
N	0.622739	-1.169889	0.189298
N	-0.683038	1.172246	-0.185905
N	0.622733	1.169873	-0.189339
C	-2.794627	-0.000010	0.000019
C	-4.719805	-1.117042	-0.383185
C	-4.719785	1.117064	0.383200
C	-5.453904	0.000017	0.000010
H	-5.210444	-2.034304	-0.701302
H	-5.210408	2.034334	0.701319
H	-6.537415	0.000026	0.000011
C	2.732008	-0.000002	-0.000014
C	3.432469	1.197116	-0.182195
C	3.432475	-1.197113	0.182190
C	4.821930	1.192897	-0.181316
H	2.879377	2.119115	-0.322734
C	4.821936	-1.192882	0.181348
H	2.879387	-2.119117	0.322712
C	5.518709	0.000011	0.000025
H	5.362027	2.123647	-0.322901
H	5.362037	-2.123627	0.322946
H	6.604473	0.000015	0.000040
N	-3.388297	1.129269	0.386726
N	-3.388318	-1.129273	-0.386706

TS11

G(water) = -1062.777032 Hartree

N	-0.634949	-0.969049	1.370860
N	0.647071	-0.959765	1.361790
C	1.251931	-1.026292	0.135533
N	0.637836	-1.789887	-0.837026
N	-0.637180	-1.799806	-0.832921
C	-1.237782	-1.038425	0.148431
C	0.652618	0.914086	-0.750937
C	-0.734865	0.882572	-0.753482
C	1.060625	2.075911	0.137781
C	0.680287	3.333210	-0.704974
C	-1.195945	2.024620	0.138410
C	-0.876314	3.298744	-0.702733
C	-0.068386	2.099763	1.181940
H	1.253678	0.619477	-1.605609
H	-1.321499	0.554143	-1.607193
H	2.091051	2.061942	0.497638
H	1.105031	3.297514	-1.711891
H	1.057503	4.235999	-0.215177
H	-2.220781	1.960005	0.509412
H	-1.301550	3.246148	-1.708794
H	-1.292001	4.182767	-0.209896
H	-0.090927	3.045212	1.733741
H	-0.055190	1.273631	1.890061
C	2.726074	-0.890696	0.086211
C	3.426137	-1.238736	-1.072102
C	3.416447	-0.376682	1.187406
C	4.805872	-1.074041	-1.125172
H	2.881832	-1.648620	-1.917101
C	4.796460	-0.214066	1.127565
H	2.861905	-0.119852	2.084364
C	5.493676	-0.560739	-0.027676
H	5.346617	-1.350102	-2.025139
H	5.329021	0.182218	1.986719
H	6.570918	-0.432604	-0.072150
C	-2.724776	-0.913590	0.095725

C	-4.597713	-0.885555	-1.175136
C	-4.687752	-0.553177	1.158188
C	-5.375837	-0.665039	-0.044203
H	-5.046472	-0.964574	-2.163016
H	-5.210823	-0.358485	2.092107
H	-6.453792	-0.570450	-0.099472
N	-3.273465	-1.003345	-1.118604
N	-3.363465	-0.673228	1.241152

TS12

G(water) = -1575.453977 Hartree

C	-1.694193	0.323965	0.986084
C	-1.914650	1.714532	0.980558
C	-3.037700	2.241976	1.615703
C	-3.940090	1.405301	2.262799
C	-3.689983	0.036004	2.323755
C	-2.569328	-0.501263	1.702799
H	-3.179495	3.319021	1.611133
H	-4.815197	1.825919	2.747766
H	-4.362095	-0.618307	2.869785
H	-2.357644	-1.562960	1.784126
C	-0.499607	-0.159932	0.317716
C	0.697741	0.094829	0.041008
C	1.688280	1.088929	-0.327128
C	2.710122	0.829588	-1.247063
C	1.515826	2.396723	0.172117
C	3.597293	1.832918	-1.611491
H	2.784442	-0.151702	-1.698393
C	2.409421	3.390402	-0.214278
C	3.460519	3.112661	-1.081178
H	4.387554	1.614915	-2.322779
H	2.250176	4.401436	0.148480
H	4.148918	3.901181	-1.368383
C	0.348691	2.737809	1.068460
H	0.330869	2.087905	1.947996
H	0.442362	3.774998	1.405874
C	1.575405	-1.922022	-0.004791
C	-0.856750	-2.063778	-0.403993
N	-0.415059	-2.683046	0.749317
N	0.837441	-2.588210	0.959560
N	-0.058067	-2.173553	-1.530632
N	1.194959	-2.098666	-1.321216
C	-2.319445	-2.085538	-0.730276
C	-4.018329	-1.149149	-1.888416
C	-4.363095	-3.011045	-0.478203
C	-4.906030	-2.074120	-1.348642
H	-4.355076	-0.382129	-2.582555
H	-4.980345	-3.778626	-0.016402
H	-5.960944	-2.065797	-1.595788
C	3.013030	-1.768702	0.317549
C	3.372421	-1.284790	1.575713
C	4.000246	-2.123409	-0.603616
C	4.714282	-1.131231	1.903191
H	2.591814	-1.025373	2.285808
C	5.341507	-1.974287	-0.269520
H	3.705331	-2.510865	-1.574135
C	5.700086	-1.472984	0.980083
H	4.992294	-0.745151	2.878821
H	6.109120	-2.253584	-0.984662
H	6.748335	-1.352925	1.236103
N	-3.068566	-3.025198	-0.159609
N	-2.721639	-1.146861	-1.588656
N	-0.949961	2.572698	0.393158
C	-1.222378	3.408253	-0.671264
O	-0.458578	4.307887	-0.967165
C	-2.478685	3.101504	-1.462366
H	-3.373041	3.449875	-0.938147
H	-2.400996	3.625709	-2.414586
H	-2.579164	2.024353	-1.628074

TS13

G(water) = -1536.157373 Hartree

C	-1.767043	0.805954	0.727138
C	-1.831721	2.196825	0.492149
C	-2.872121	2.953302	1.009032
C	-3.854967	2.342853	1.783644
C	-3.771634	0.981010	2.062852
C	-2.731435	0.214137	1.549094
H	-2.883052	4.020525	0.813490
H	-4.668576	2.934663	2.190934
H	-4.513116	0.510355	2.700701
H	-2.647410	-0.836353	1.809960
C	-0.615533	0.098406	0.183518
C	0.601099	0.166193	-0.124205
C	1.681040	0.987957	-0.652512
C	2.700443	0.500962	-1.474792
C	1.628412	2.372282	-0.383053
C	3.683890	1.360385	-1.951603

H	2.705645	-0.544365	-1.758131
C	2.630222	3.219705	-0.837558
C	3.666791	2.712278	-1.616770
H	4.468789	0.967080	-2.589540
H	2.584209	4.276171	-0.590673
H	4.448564	3.373864	-1.976064
C	0.442597	2.840858	0.409726
N	-0.777090	2.786257	-0.268490
C	-0.869003	2.598384	-1.714840
H	-0.903382	1.538767	-1.998799
H	-1.782730	3.087009	-2.063237
H	-0.015501	3.075546	-2.198368
O	0.514790	3.128949	1.583401
C	1.287813	-1.866084	0.217515
C	-1.141342	-1.878394	-0.214286
N	-0.769042	-2.313147	1.042495
N	0.484334	-2.289085	1.265199
N	-0.343022	-2.254797	-1.280128
N	0.910629	-2.249969	-1.055323
C	-2.592249	-1.813521	-0.576578
C	-4.158861	-0.946979	-1.953660
C	-4.727196	-2.455058	-0.227419
C	-5.150173	-1.653233	-1.280234
H	-4.399147	-0.298700	-2.793359
H	-5.431726	-3.050140	0.349413
H	-6.193782	-1.583974	-1.563165
C	2.728291	-1.764677	0.547783
C	3.106193	-1.061348	1.692361
C	3.696268	-2.373676	-0.252943
C	4.451683	-0.943969	2.020450
H	2.340645	-0.597936	2.309047
C	5.040640	-2.258437	0.082941
H	3.385966	-2.929983	-1.132374
C	5.419938	-1.539311	1.214780
H	4.744275	-0.384437	2.903110
H	5.793904	-2.734502	-0.537243
H	6.470888	-1.446677	1.471329
N	-3.447375	-2.542124	0.135487
N	-2.874865	-1.021326	-1.612015

Materials and Methods

Small molecule reaction kinetics

The reaction rates between diaryltetrazine **13** and 5-norbornene-2-*endo*-acetic acid **12** were measured under *pseudo*-first order reaction conditions by using 10-80 fold excess 5-norbornene-2-*endo*-acetic acid **12** in water/methanol mixtures. Stock solutions of diaryltetrazine **13** were prepared in 9/1 water/methanol (0.1 mM) and 5-norbornene-2-*endo*-acetic acid **12** in methanol (1, 3, 5 and 8 mM). Equal volumes (0.5 mL each) of stock solutions, resulting final concentration 0.05 mM of tetrazine and 0.5, 1.5, 2.5, 4 mM of norbornene were mixed by maintaining MeOH:H_2O (55:45). The exponential decay in UV absorbance of tetrazine **13** at 320 nm was measured over time. The *pseudo*-first order reaction kinetics for DIBAC **10** and benzyl azide were measured by following the exponential decay in UV absorbance of DIBAC **10** at 310 nm upon reaction with 20-100 fold excess benzyl azide in methanol-water (55:45). Stock solutions, 0.05 mM of DIBAC **10** in methanol and 1, 2, 3, 4 and 5 mM of benzyl azide in 9/1 water/methanol were prepared. The *pseudo*-first order reaction kinetics for BARAC **6** and benzyl azide were measured by following the exponential decay in UV absorbance of BARAC **6** at 306 nm upon reaction with 20-100 fold excess benzyl azide in acetonitrile-water (1:1). Spectra were recorded on a Shimadzu UV-Vis-NIR spectrometer. All the data were recorded at 23 ℃ using spectral band-width (SBW) = 1.0 nm, path length = 1.0 cm with increment in data point collection at 2 seconds. The observed rate constants k′ were determined by fitting each data set to a single-exponential equation. The k′ values were then plotted against the concentration of norbornene **12** or benzyl azide and subjected to a linear fit to yield a plot with slope k_2, the second order rate constant. Each kinetic experiment was performed in triplicate and the three k_2 values were averaged. All data was processed on Origin8 software program. Due to direct overlap in UV absorbance of DIBAC or BARAC with *N*-phenyl sydnone, the rate constants were calculated from competition experiments between *N*-phenyl sydnone and benzyl azide with DIBAC or BARAC via NMR spectroscopy. On the basis of the product ratios of NMR competition experiments and the determined rate constants for the benzyl azide cycloadditions of DIBAC or BARAC, the rate constants for the *N*-phenyl sydnone cycloadditions of DIBAC or BARAC were obtained.

Cross-reactivity kinetics

The cross reactivity of DIBAC **10** with diaryltetrazine **13** and 5-norbornene-2-*endo*-acetic acid **12** with sydnone **1** was monitored via NMR spectroscopy at room temperature over a period of 24 h. No reaction was observed between either reaction pair after 24 h.

Preparation of protein conjugation

Bovine serum albumin (BSA) and Ovalbumin from chicken egg white (OVA) conjugates were prepared by treating the appropriate protein with the succinimidyl esters of either DIBAC (DIBAC-NHS) (CP-2033, Conju-Probe, San Diego, CA) or 5-norbornene-2-*endo*-acetic acid (Nor-NHS) (Sigma-Aldrich) using standard coupling conditions. In short, BSA and OVA (0.5 mL of a 20 mg/mL solution in PBS pH 7.4) were combined with DIBAC-NHS and Nor-NHS (100 µL of 20 mM solution in DMSO) respectively. The reaction mixture was incubated at 37 ℃ with shaking for 3 h then allowed to stand at rt for 12 h. Column purification was performed to remove excess DIBAC-NHS and Nor-NHS from the protein-conjugates. The reaction mixture was loaded onto a Zeba™ Spin Desalting Column, pre-equilibrated with 10 mL PBS, and the eluted fractions were collected.

In-gel fluorescence analysis of BSA and Ova conjugates

Purified DIBAC- and norbornene-modified BSA and OVA protein conjugate samples (20 µL each of a 2 mg/mL solution in PBS, pH 7.4) were treated with **Syd-630** (5 µL of 5 mM solution in DMSO), **Tz-504** (5 µL of 5 mM solution in DMSO) or both reagents. For dual fluorescent labeling in tandem, the DIBAC- and norbornene-modified protein conjugates were combined 1:1 prior to labeling with fluorophores. The fluorophore labeling reactions were conducted for 1-60 min. After completion of 60 min, proteins were analyzed by SDS polyacrylamide gel electrophoresis. The in-gel fluorescence labeling was observed by scanning of gels with GE Typhoon 9410 and 9400 TRIO+ Variable Mode Imager. **Syd-630** fluorescence was measured with a 633 nm excitation wavelength and 670 BP 30 emission filter. **Tz-504** was measured with a 488 excitation wavelength and 520 BP 40 emission filter. The gels were rinsed with destaining buffer (50 % DI water, 40 % EtOH, 10 % AcOH) for 3 h. The protein loading was confirmed by staining the gel with Coomassie Brilliant Blue.

Chemistry-General procedures

All chemicals and reagents were purchased from commercial sources and used without further purification. BODIPY-FL-NHS ester and BODIPY 630/650 dyes were purchased from Life technologies. DIBAC **10** was purchased from Click Chemistry Tools (A103). All deuterated solvents were purchased from Cambridge Isotope Laboratories. Unless otherwise noted, reactions were carried out in oven-dried glassware under an atmosphere of argon using commercially available anhydrous solvents. Solvents used for extractions and chromatography were not anhydrous. Reactions and chromatography fractions were analyzed by thin-layer chromatography (TLC) using Merck precoated silica gel 60 F_{254} glass plates (250 μm) and visualized by ultraviolet irradiation, potassium permanganate stain or ninhydrin stain. Flash column chromatography was performed using E. Merck silica gel 60 (230−400 mesh) with compressed air. NMR spectra were recorded on ARX 400 (400 Hz) or ARX 500 (500 MHz) spectrometers. Chemical shifts are reported in parts per million (ppm, δ) using the residual solvent peak as the reference. The coupling constants, *J*, are reported in Hertz (Hz), and the multiplicity identified as the following: br (broad), s (singlet), d (doublet), t (triplet), q (quartet), and m (multiplet). High-resolution electrospray mass spectrometry data was collected with a Waters LCT Premier XE time-of-flight instrument controlled by MassLynx 4.1 software. Samples were dissolved in methanol and infused using direct loop injection from a Waters Acquity UPLC into the Multi-Mode Ionization source. HPLC purifications were performed on a Knauer Smartline HPLC system with inline Knauer UV (254 nm) detector. Semi-preprative HPLC was performed using Phenomenex reverse-phase Luna column (10 × 250 mm, 5 μm) with a flow rate of 4 mL/min. Final purity of compounds was determined by analytical HPLC analysis performed with a Phenomenex reverse-phase Luna column (4.6 × 250 mm, 5 μm) with a flow rate of 1 mL/min. Compounds were identified by UV absorbance at 254 nm. All chromatograms were collected by a GinaStar (raytest USA, Inc.; Wilmington, NC, USA) analog to digital converter and GinaStar software (raytest USA, Inc.).

Synthetic Procedures

Synthesis of sydnone derivatives

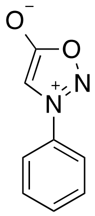

1

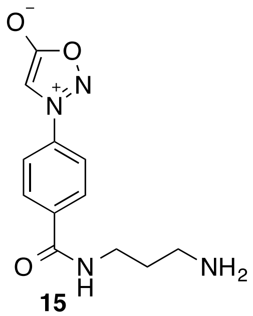

15

Sydnones **1** and **15** were synthesized according to literature procedure.[6] The ^{1}H and ^{13}C NMR spectroscopic data were consistent with previously reported values.

3-phenyl-1,2,3-oxadiazol-3-ium-5-olate (1): Yield 85 %. HRMS (ESI) *m/z* calcd for $C_8H_6N_2O_2$ [M+H] 163.0508, found 163.0501.

3-(4-((3-aminopropyl)carbamoyl)phenyl)-1,2,3-oxadiazol-3-ium-5-olate (**15**)**:** Yield = 45 %. HRMS (ESI) *m/z* calcd for $C_{12}H_{14}N_4O_3$ [M+Na] 285.0164, found 285.0164.

Synthesis of diaryltetrazine derivatives

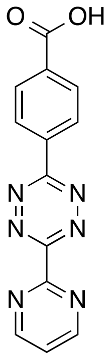

16

4-(6-(Pyrimidin-2-yl)-1,2,4,5-tetrazin-3-yl)benzoic acid (**16**) was prepared according to the literature procedure.[7] Tetrazine **16** was purified by hot DMF filtration, the process was repeated three times in order to remove symmetrical byproduct 4,4'-(1,2,4,5-tetrazine-3,6-diyl)dibenzoic acid. Yield 20 %. The ^{1}H and ^{13}C NMR spectroscopic data were identical to that reported previously in the literature.[7] HRMS (ESI) *m/z* calcd for $C_{13}H_8N_6O_2$ [M-H] 279.0640, found 279.0632.

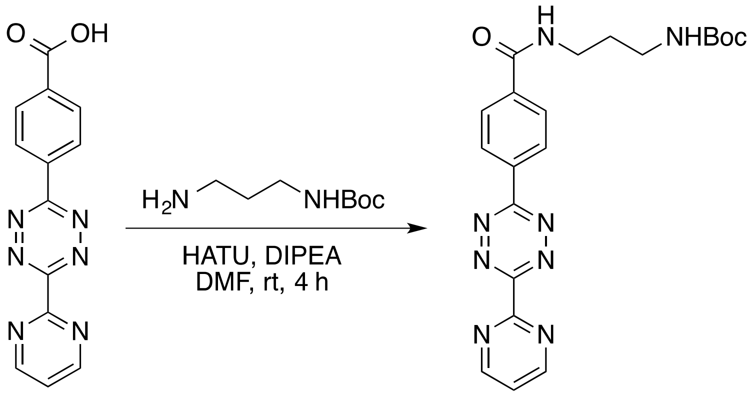

16 13

Tert-butyl(3-(4-(6-(pyrimidin-2-yl)-1,2,4,5-tetrazin-3-yl)benzamido)propyl)carbamate (13). HATU (95 mg, 0.25 mmol) was added to a stirring solution of tetrazine **16** (70 mg, 0.25 mmoles) and DIPEA (60 µL, 0.35 mmoles) in DMF (3 mL) at 0 ℃. After 5 min at 0 ℃ *N*-boc-1,3-propanediamine (50 µL, 0.27 mmoles) was added. The reaction mixture was warmed to room temperature and stirred for 4 h. The crude reaction mixture was washed with 20 % aqueous citric acid (40 mL) and extracted with ethyl acetate (3 x 30 mL). The combined organic fractions were washed with brine solution (50 mL), dried over sodium sulfate, and concentrated under reduced pressure. The crude reaction mixture was purified by silica gel column chromatography (9:1 DCM:MeOH) to yield tetrazine **13** as pink solid (58 mg, 55 %). ^{1}H NMR (400 MHz, $CDCl_3$) δ 9.14 (d, *J* = 4.9 Hz, 2H), 8.82 (d, *J* = 8.5 Hz, 2H), 8.13 (d, *J* = 8.3 Hz, 2H), 7.65 (s, 1H), 7.60 (t, *J* = 4.9 Hz, 1H), 4.88 (s, 1H), 3.56 (dd, *J* = 12.1, 6.1 Hz, 2H), 3.30 (dd, *J* = 11.9, 6.2 Hz, 2H), 1.79-1.72 (m, 2H), 1.47 (s, 9H). ^{13}C NMR (125 MHz, $CDCl_3$) δ 166.34, 164.16, 163.18, 159.49, 158.48, 157.25, 138.83, 133.73, 129.00, 128.08, 122.60, 79.90, 42.25, 36.10, 30.17, 28.42. HRMS (ESI) *m/z* calcd for $C_{21}H_{24}N_8O_3$ [M+H] 437.0768, found 437.0767.

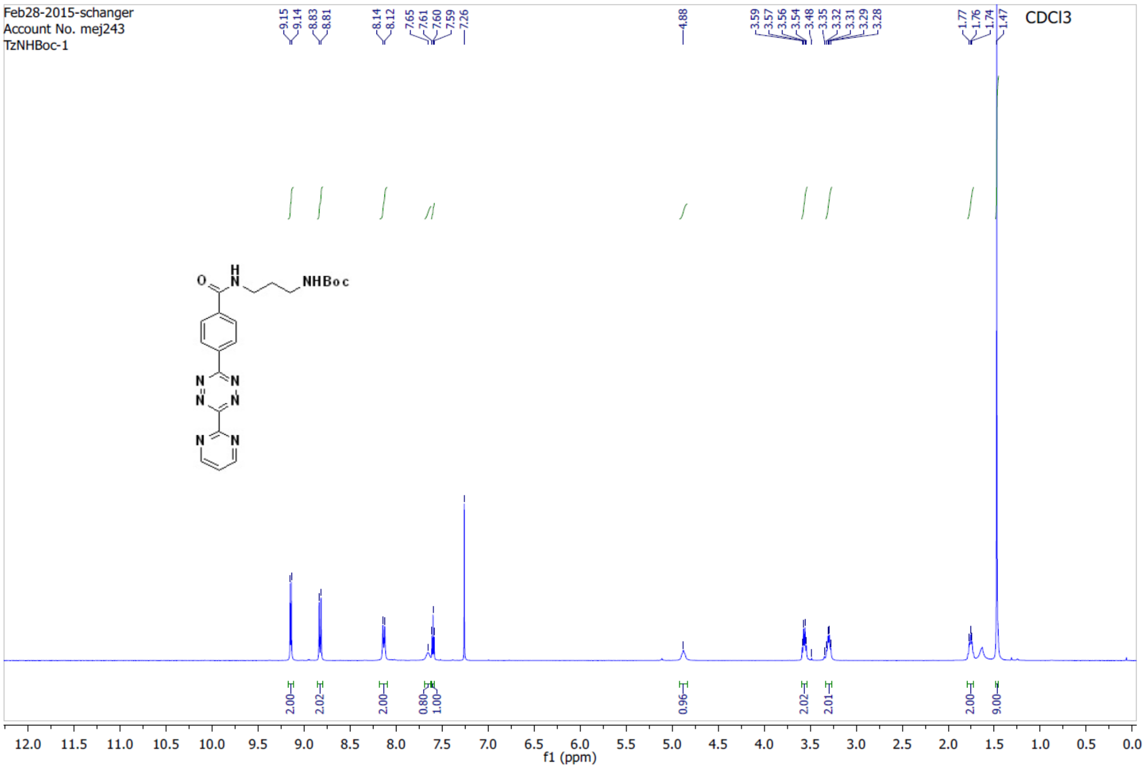
Feb28-2015-schanger
Account No. mej243
TzNHBoc-1
CDCl3
NHBoc
f1 (ppm)

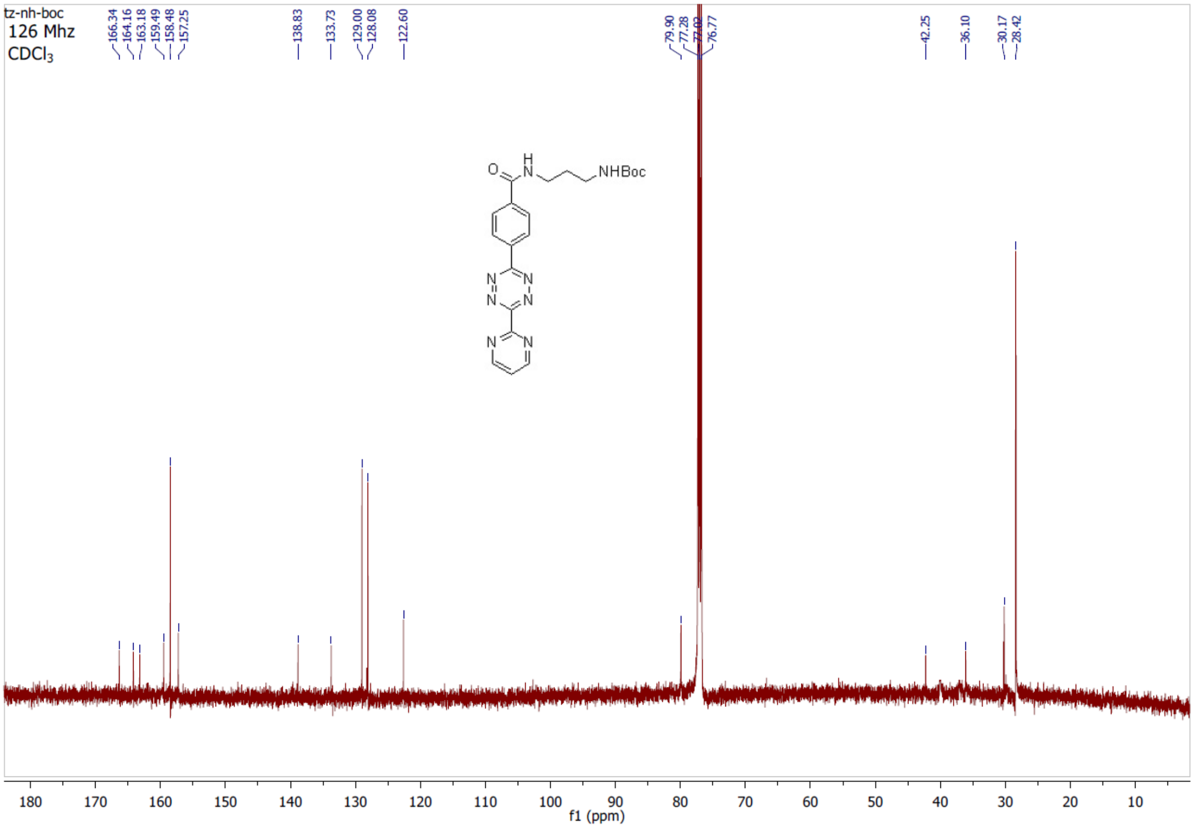
tz-nh-boc
126 Mhz
$CDCl_3$
NHBoc
f1 (ppm)

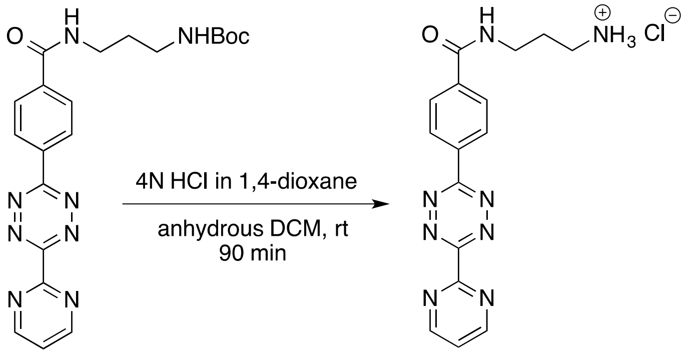

13 **17**

***N*-(3-Aminopropyl)-4-(6-(pyrimidin-2-yl)-1,2,4,5-tetrazin-3-yl)-benzamide hydrochloride (17).** To a stirred solution of compound **13** (20 mg, 0.045 mmol) in dry dichloromethane (5 ml) a 4N HCl solution in 1,4-dioxane (2 ml) was added and the reaction mixture was allowed to stir for 90 min at rt. Consumption of **13** was observed by TLC analysis and the reaction mixture was concentrated to dryness under reduced pressure, to give desired tetrazine **17** as the HCl salt (15 mg, 92%) which was used without further purification. ^{1}H NMR (400 MHz, DMSO- d_6) δ 9.17 (d, *J* = 4.9 Hz, 2H), 8.95 (t, *J* = 5.7 Hz, 1H), 8.64 (d, *J* = 8.6 Hz, 2H), 8.15 (d, *J* = 8.6 Hz, 2H), 7.92 (s, 2H), 7.81 (t, *J* = 4.9 Hz, 1H), 3.36 (q, *J* = 6.5 Hz, 2H), 2.84 (dd, *J* = 13.8, 6.5 Hz, 2H), 1.88-1.80 (m, 2H). ^{13}C NMR (125 MHz, DMSO-d_6) δ 166.57, 163.32, 159.36, 159.01, 158.24, 138.39, 134.44, 128.75, 127.89, 123.58, 37.40, 36.93, 27.73. HRMS (ESI) *m/z* calcd for $C_{16}H_{16}N_8O$ [M+H] 337.1352, found 337.1359.

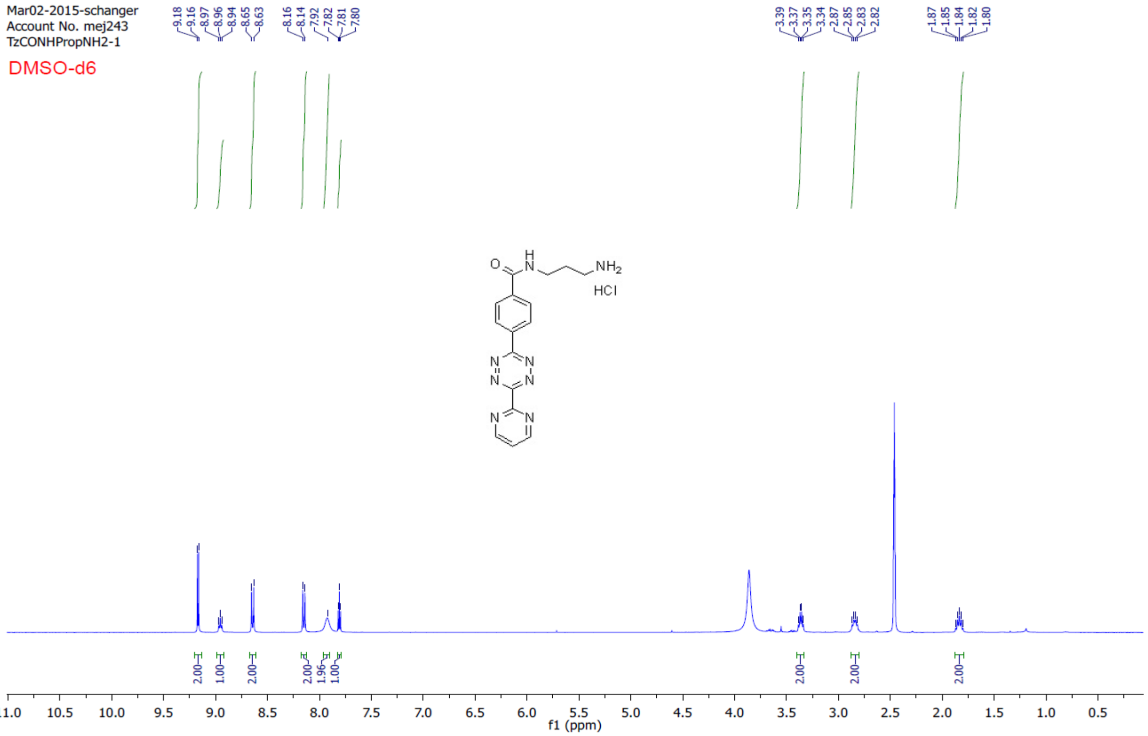
Mar02-2015-schanger
Account No. mej243
TzCONHPropNH2-1
DMSO-d6
9.18
9.16
8.97
8.96
8.94
8.65
8.63
8.16
8.14
7.92
7.82
7.81
7.80
3.39
3.37
3.35
3.34
2.87
2.85
2.83
2.82
1.87
1.85
1.84
1.82
1.80
HCl
2.00
1.00
2.00
2.00
1.96
1.00
2.00
2.00
2.00
f1 (ppm)

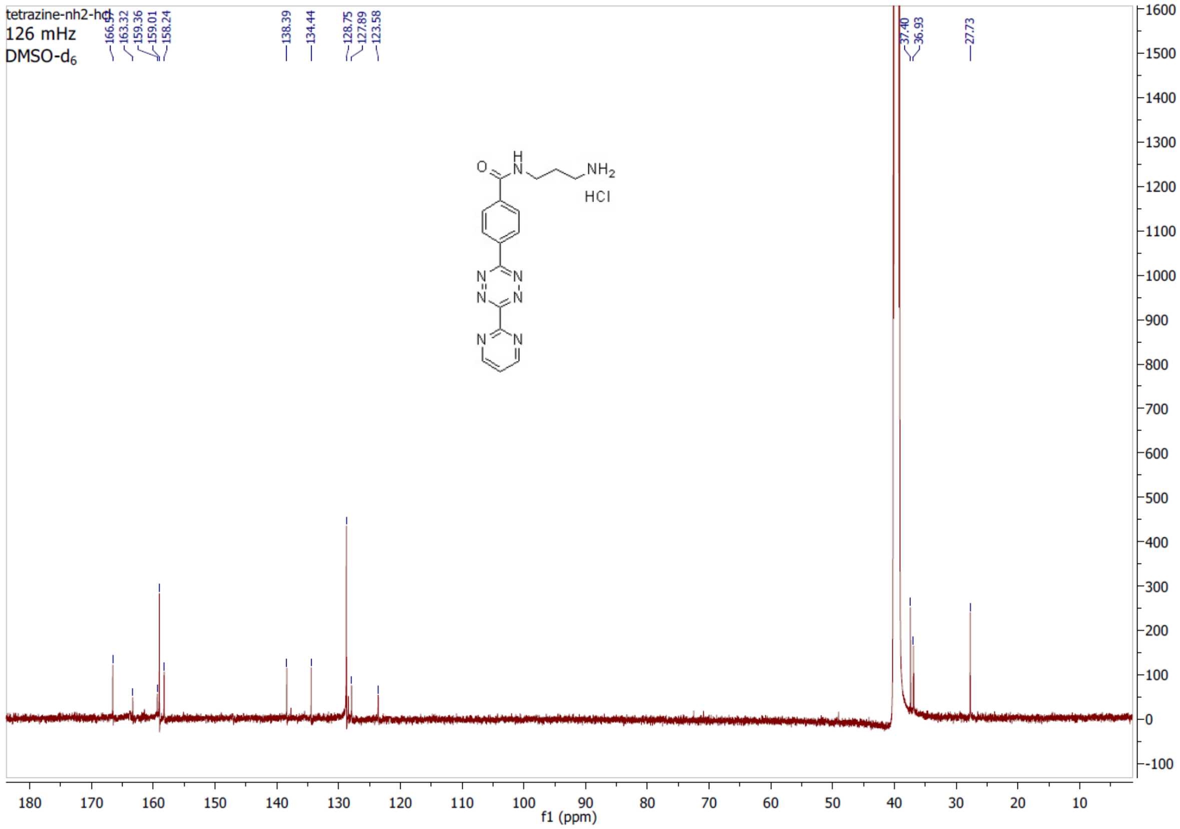
tetrazine-nh2-hcl
126 mHz
DMSO-d6
166.57
163.32
159.36
159.01
158.24
138.39
134.44
128.75
127.89
123.58
37.40
36.93
27.73
HCl
f1 (ppm)

Synthesis of BARAC

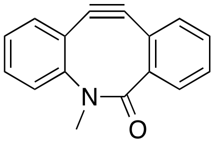

6
BARAC

Synthesis of BARAC **6** was carried out following the literature reported protocol.[8] All the intermediates were characterized and in agreement with the previously reported data. The final product was purified by semi-preparative HPLC (30% to 75% CH_3CN in water over 35 min) and the ^{1}H and ^{13}C NMR spectroscopic data were identical to that previously reported in the literature. [8] Yield 40 % (final step). HRMS (ESI) *m/z* calcd for $C_{16}H_{11}NO$ [M+H] 234.0919, found 234.0919.

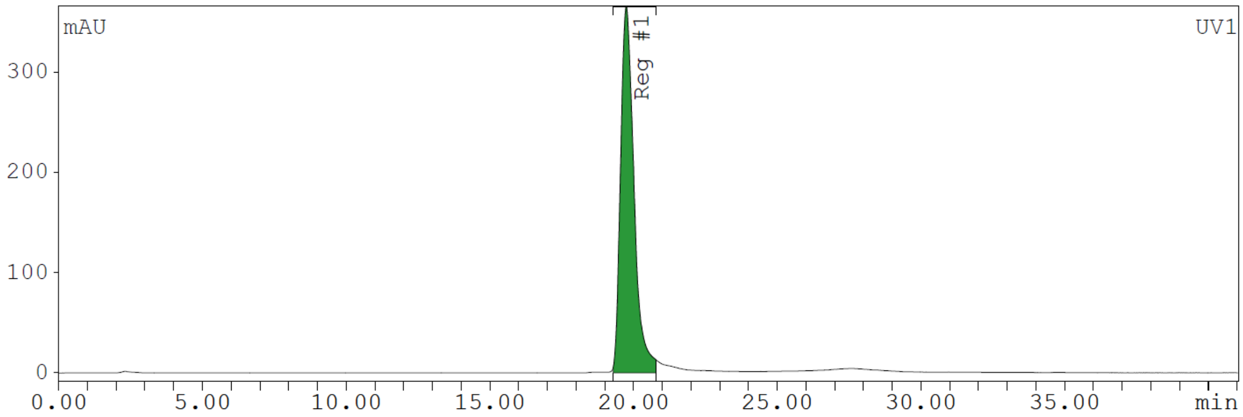

Figure S1. HPLC trace of purified BARAC **6** using a gradient of 30% to 75% acetonitrile in water over 35 min.

Synthesis of small molecule fluorophore conjugates

Sydnone-BODIPY630 (Syd-630) and Tetrazine-BODIPY504 (Tz-504)

To a solution of the amine reactive BODIPY NHS dye (3 µmol) in DMF, the corresponding sydnone **15** (15 µmol) or tetrazine **17** (6 µmol) and *N*,*N*-diisopropylethylamine (5 µmol) were added. The reaction mixtures were allowed to stir at room temperature for 20 h in the dark. DMF was removed under reduced pressure and the dye-conjugates were purified by semi-preparative reverse phase HPLC (10% to 85% CH_3CN in water over 35 minutes, 3mL/min flow rate). The identity and purity of the conjugates were confirmed by analytical HPLC and HRMS.

Syd-630 HRMS (ESI) *m/z* calcd for $C_{41}H_{40}BF_2N_7O_6S$ [M+H] 808.029, found 808.0386.

Tz-504 HRMS (ESI) *m/z* calcd for $C_{30}H_{29}BF_2N_{10}O_2$ [M+Na] 633.2434, found 633.2450.

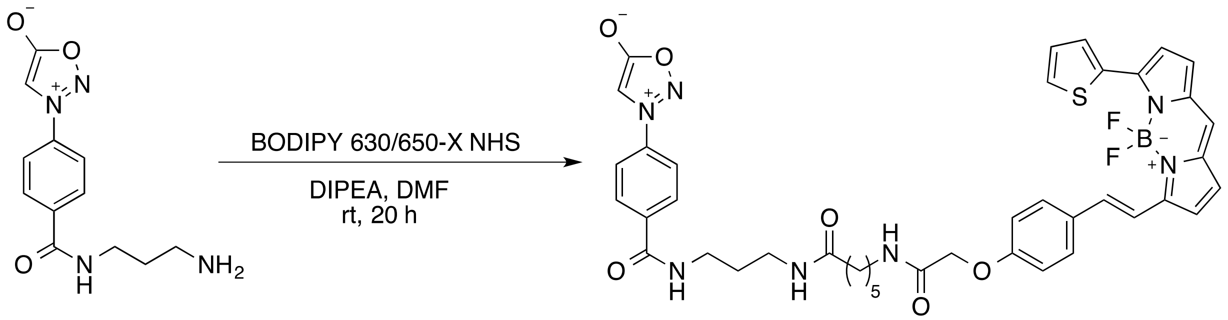

15 **Syd-630**

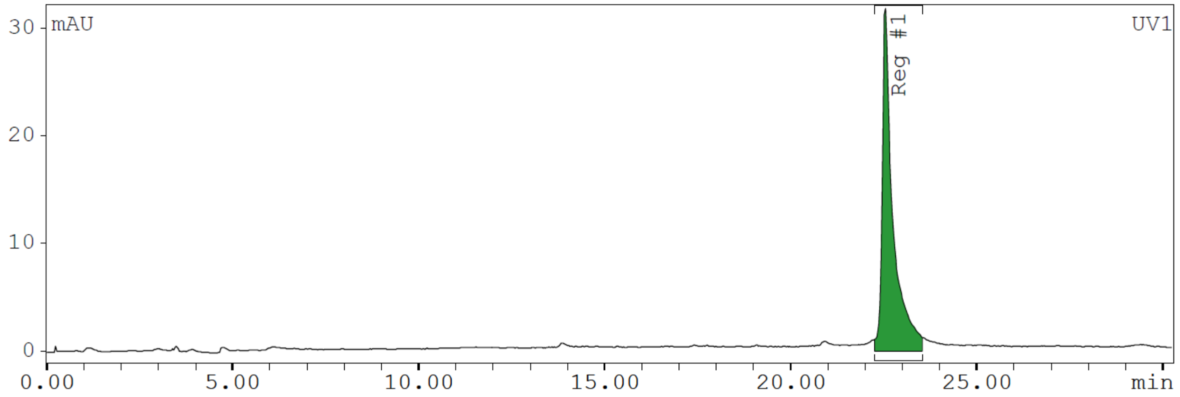

Figure S2. HPLC trace of purified **Syd-630** using a gradient of 10 % to 90 % acetonitrile in water over 30 min.

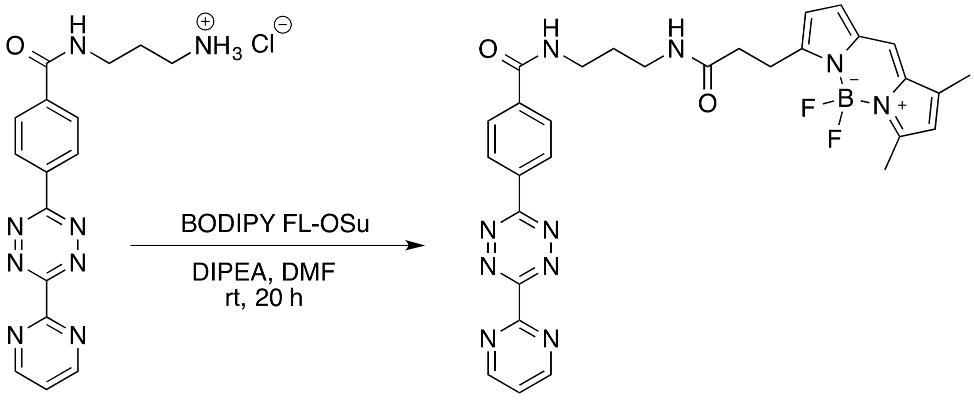

17 **Tz-504**

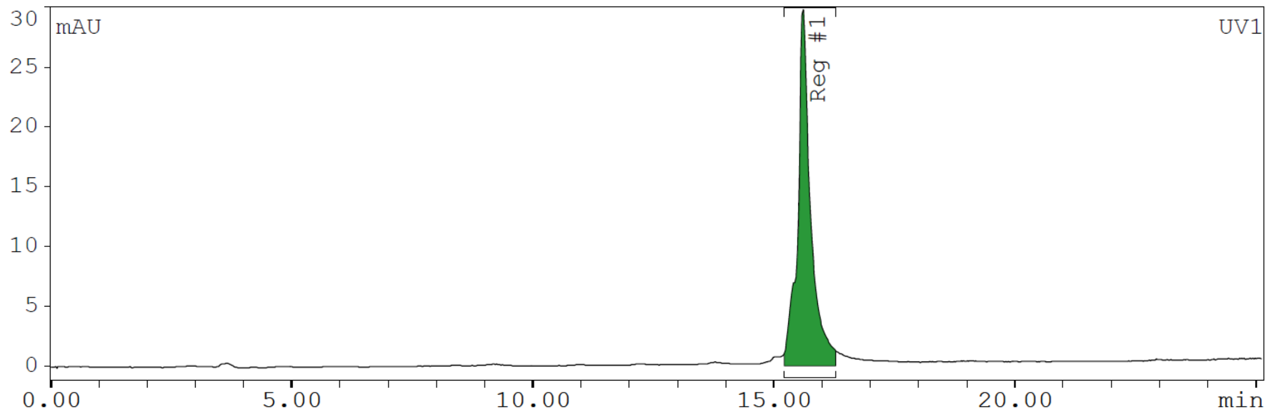

Figure S3. HPLC trace of purified **Tz-504** using a gradient of 20 % to 90 % acetonitrile in water over 25 min.

A

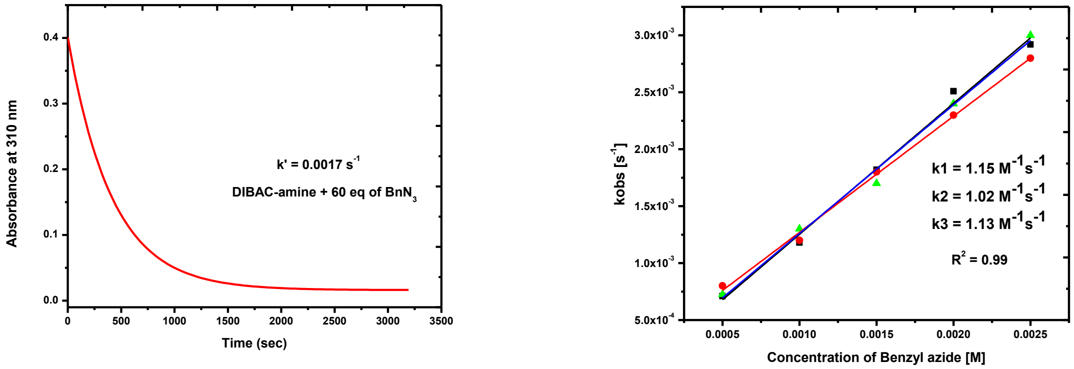

B

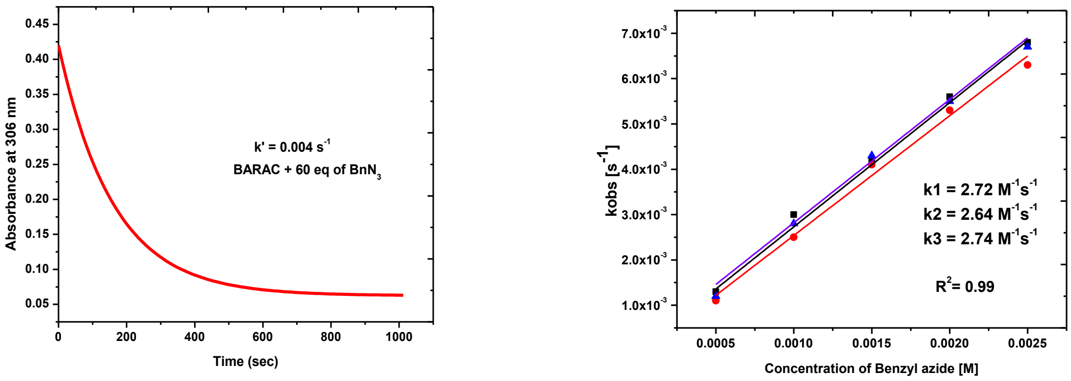

Figure S4. Determination of the second-order rate constants for the reactions of DIBAC **10** and BARAC **6** with benzyl azide. Exponential decay in UV absorbance at 310 nm of 60 eq of benzyl azide reacting with 1 eq of DIBAC **10** (A, left panel) and exponential decay in UV absorbance at 306 nm of 60 eq of benzyl azide reacting with 1 eq of BARAC **6** (B, left panel); by fitting the data to a single exponential equation, k′ values were determined. Plot of observed rate constants k′ vs. the concentration of benzyl azide (right panels). The second-order rate constant k_2 is calculated from the slope of the linear regression line. The calculated rate constant for DIBAC-benzyl azide cycloaddition in MeOH:H_2O (55:45) was 1.10 $\pm$0.05 M^{-1} s^{-1} and the rate constant for BARAC-benzyl azide cycloaddition in CH_3CN:H_2O (1:1) was 2.70 $\pm$0.11 M^{-1} s^{-1}.

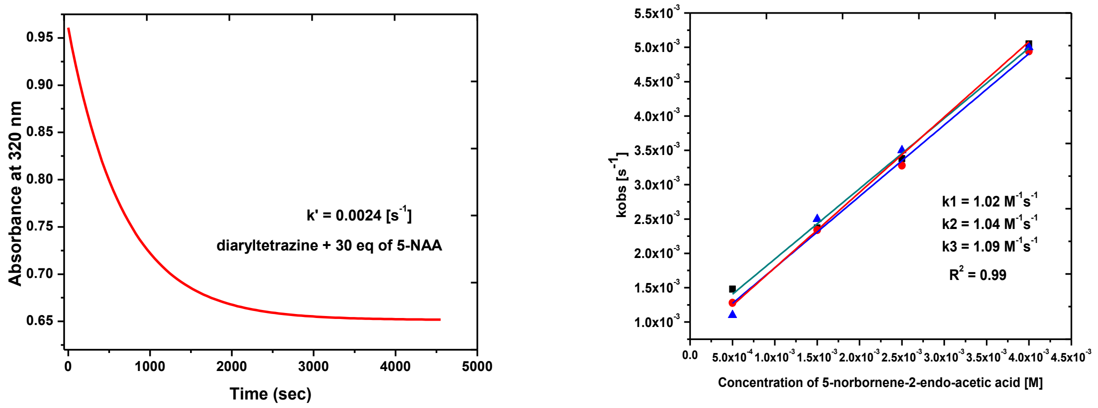

Figure S5. Determination of the second-order rate constant k_2 of diaryltetrazine **13** and 5-norbornene-2-*endo*-acetic acid **12** via the same method as described in **Figure S4**. The calculated rate constant was 1.05 ± 0.04 M^{-1} s^{-1}.

General Procedure for reaction rate determination by NMR competition experiments:

To a solution of benzyl azide (100 μmol) and phenyl sydnone **1** (100 μmol) in solvent, DIBAC **10** (10 μmol) or BARAC **6** (10 μmol) was added. After 4 hrs of shaking, the solvents were evaporated and crude material was dissolved in CD_3CN. A ^1H NMR spectrum was recorded to determine the ratio of the cycloadducts derived from benzyl azide and phenyl sydnone. DIBAC **10**-benzyl azide cycloaddition was carried out in $CD_3OD:D_2O$ (55:45) and BARAC-benzyl azide cycloaddition was conducted in $CD_3CN:D_2O$ (1:1).

Competition experiment for BARAC + Benzyl azide + Phenyl sydnone in $CD_3CN:D_2O$ (1:1)

δ 3.14 s, 1 × CH_3 adduct with benzyl azide: integration is 3.00

δ 3.20 s, 3.20 s, 2 × CH_3 adducts with phenyl sydnone **1**: integration is 3.25

Ratio of BARAC + benzyl azide : BARAC + phenyl sydnone = (3.00/3) : (3.25/6) = 1 : 0.54

Rate constant for BARAC + benzyl azide = 2.70 $M^{-1}s^{-1}$

Calculated rate constant for BARAC + phenyl sydnone = 0.54 × 2.70 = 1.46 $M^{-1}s^{-1}$

Competition spectrum is shown in Figure S12.

Competition experiment for DIBAC + Benzyl azide + Phenyl sydnone in $CD_3OD:D_2O$ (55:45)

δ 5.98 d, 1H; δ 5.90 d, 0.75H, adduct with phenyl sydnone **1**: integration is 0.80+0.63 = 1.43

δ 5.52 d, 1H, adduct with benzyl azide: integration is 1.00

Ratio of DIBAC + benzyl azide : DIBAC + phenyl sydnone = (1.00/1) : (1.43/1.75) = 1 : 0.82

Calculated rate constant for DIBAC + benzyl azide = 1.10 $M^{-1}s^{-1}$

Calculated rate constant for DIBAC + phenyl sydnone = 0.82 $\times$ 1.10 = 0.902 $M^{-1}s^{-1}$

Competition spectrum is shown in Figure S13.

High-Resolution Mass Spectroscopy of cycloaddition adducts

Cycloadduct **11**: DIBAC **10** with phenyl sydnone **1**, HRMS (ESI) *m/z* calcd for $C_{25}H_{23}N_4O$ (M+H) 395.0754, observed 395.0771.

Cycloadduct **7**: BARAC **6** with phenyl sydnone **1**, HRMS (ESI) *m/z* calcd for $C_{23}H_{18}N_3O$ (M+H) 352.0250, observed 352.0276.

Cycloadduct: DIBAC **10** and benzyl azide, HRMS (ESI) *m/z* calcd for $C_{25}H_{24}N_5O$ (M+H) 410.1981, observed 410.1985.

Cycloadduct: BARAC **6** with benzyl azide, HRMS (ESI) *m/z* calcd for $C_{23}H_{19}N_4O$ (M+H) 367.1559, observed 367.1561.

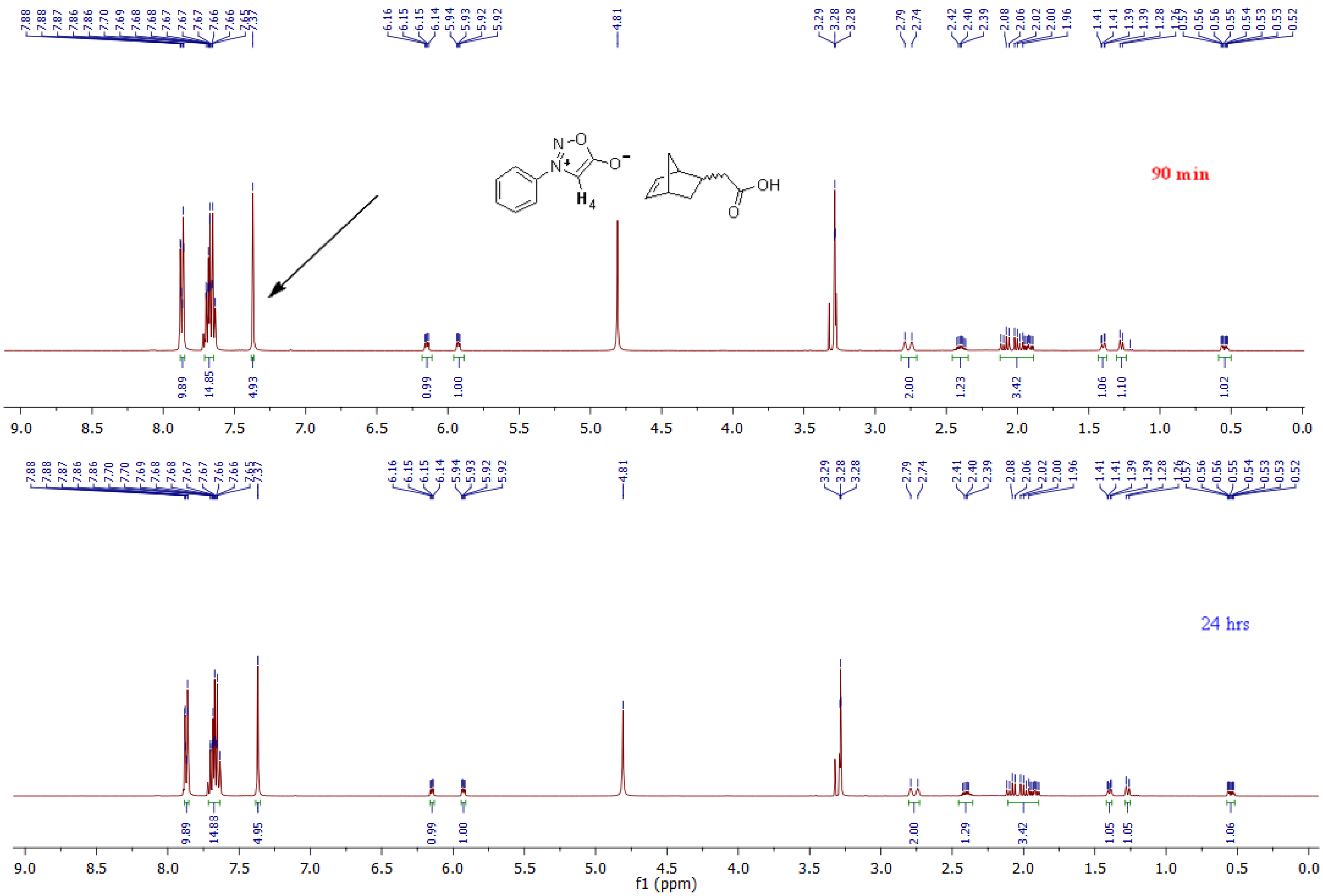

Figure S6. Reactivity of norbornene acetic acid **12** with excess phenyl sydnone **1** was assessed by ^{1}H NMR (400 MHz) in MeOD over a period of 24 hours. The **H_4** signal at δ 7.37 ppm of sydnone was taken as reference and indicated in both the spectra

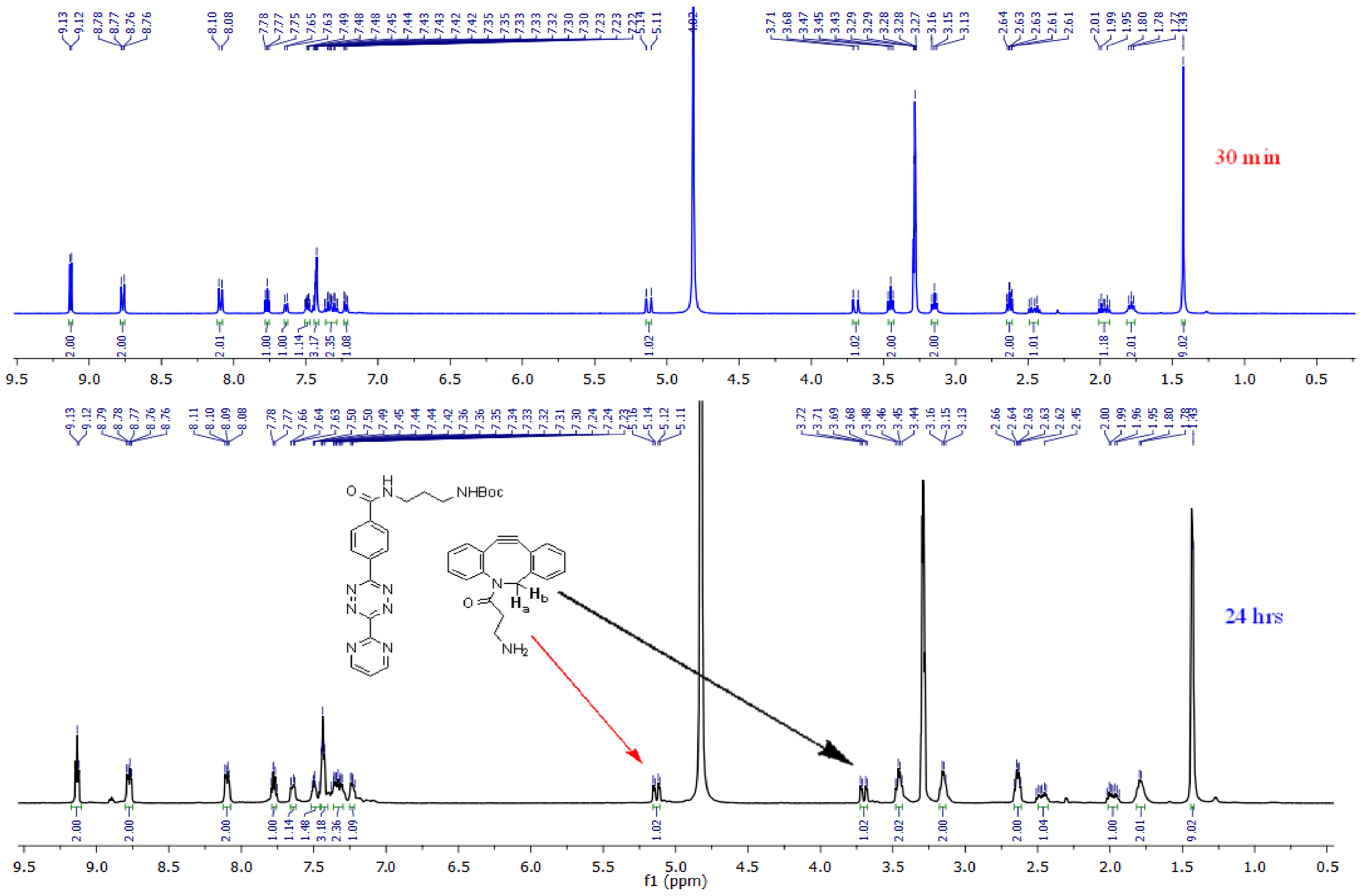

Figure S7. Reactivity of DIBAC **10** and diaryltetrazine **13** was assessed by ^{1}H NMR (400 MHz) in MeOD over a period of 24 hours. The doublets of CH_2 (**H$_a$** and **H$_b$**) of DIBAC **10** indicated in both spectra were taken as reference.

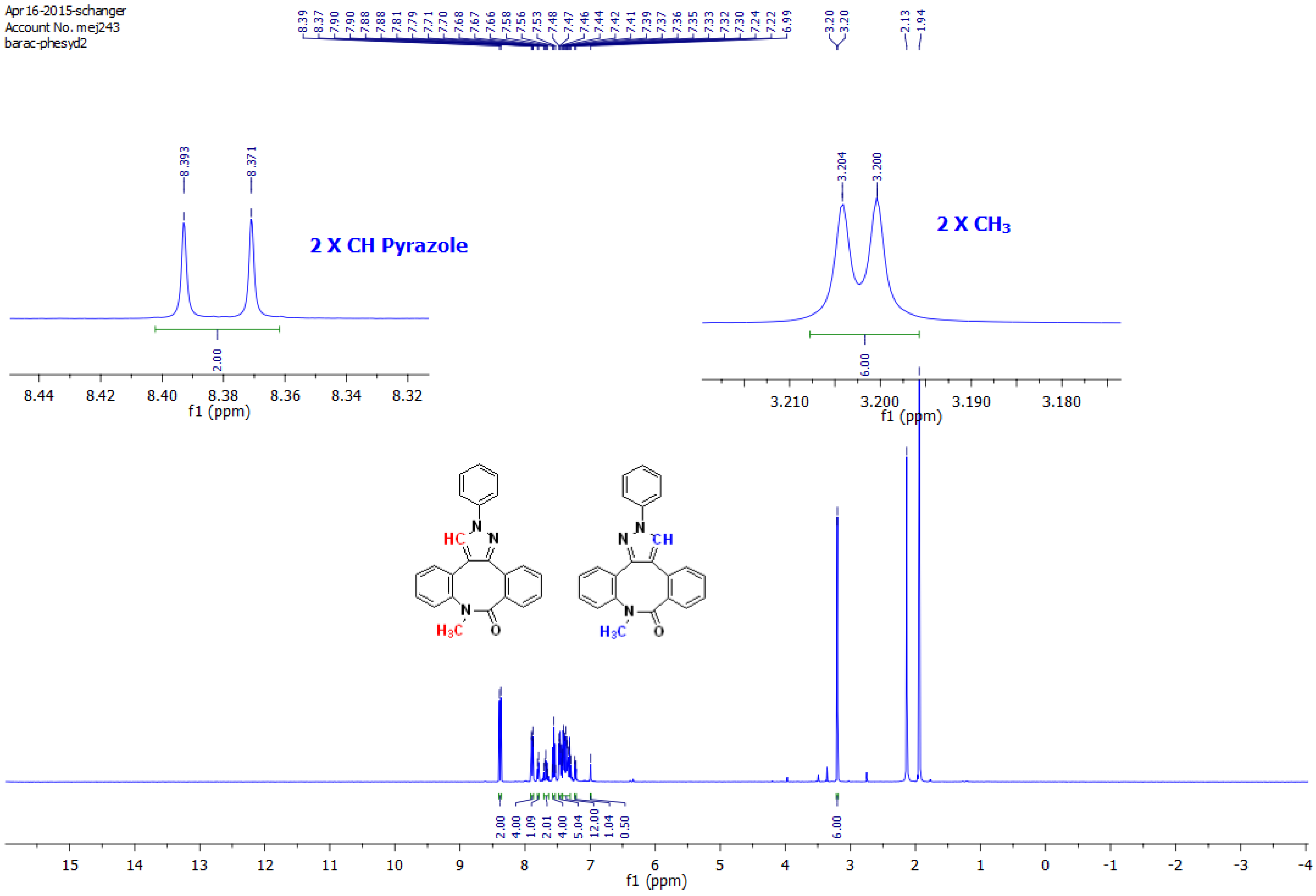

Figure S8. The ^{1}H NMR (400 MHz) spectrum of cycloadducts **7** in CD_3CN.

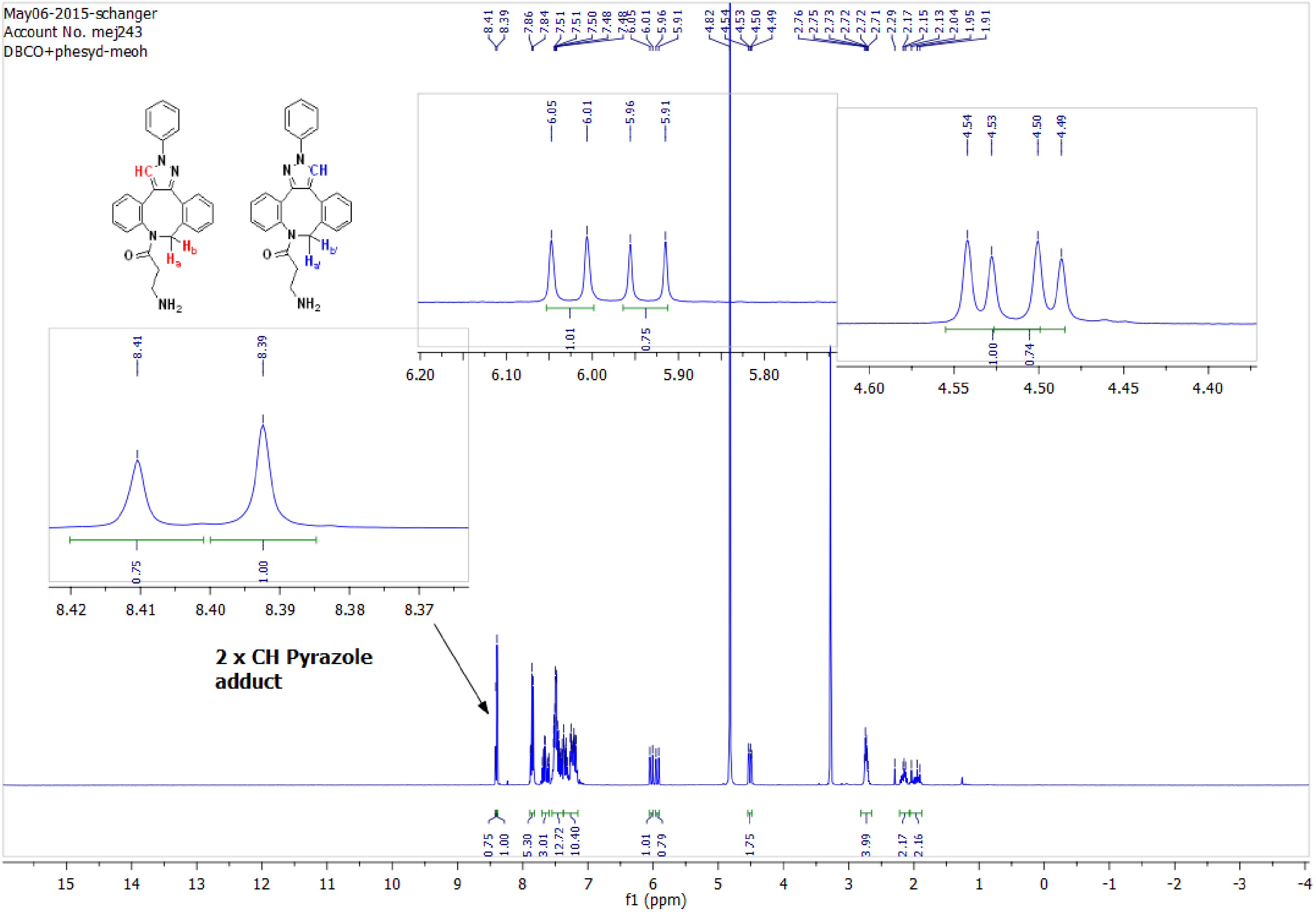

Figure S9. The ^{1}H NMR (400 MHz) spectrum of cycloadducts **11** in MeOD.

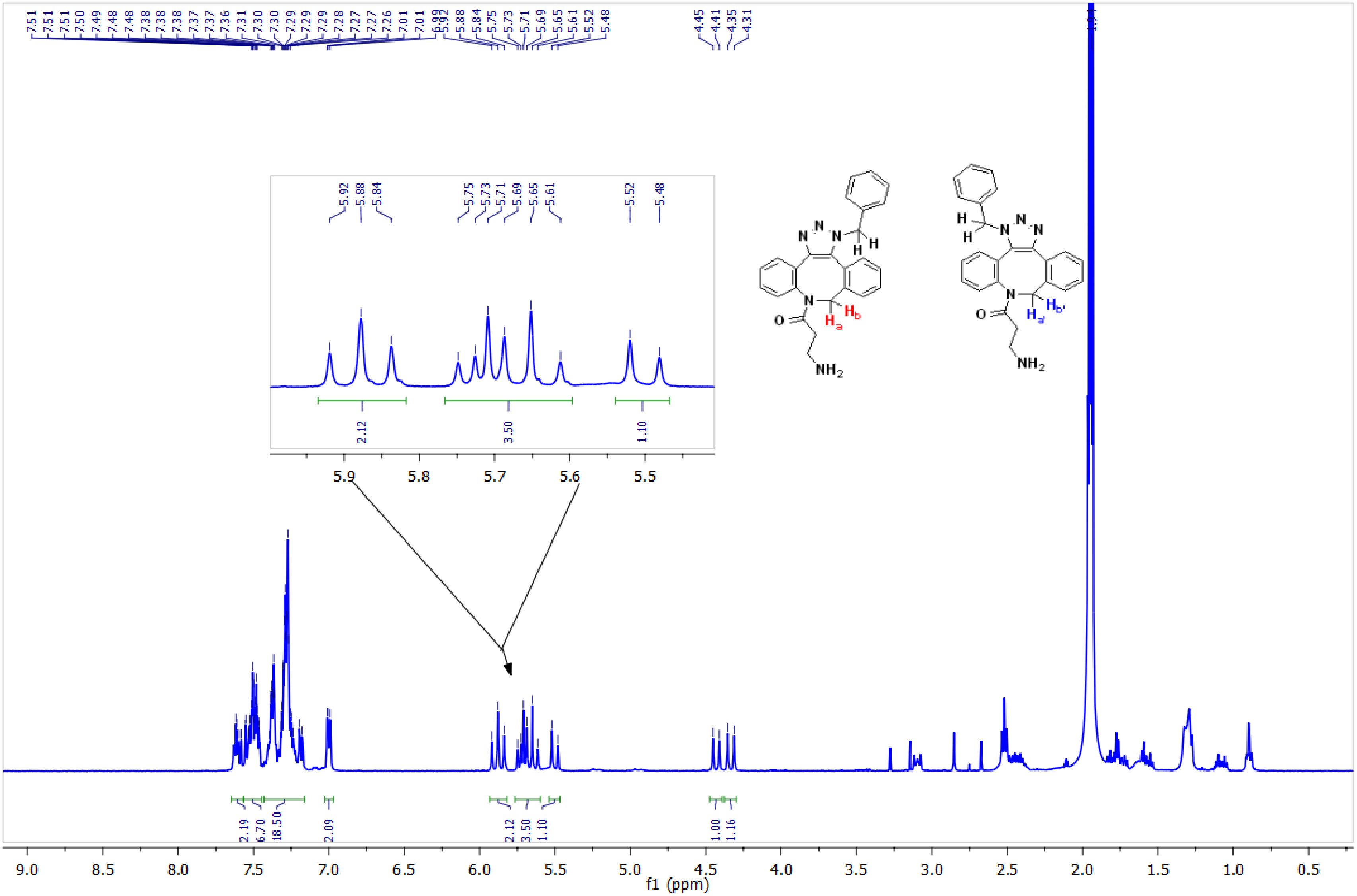

Figure S10. The ^{1}H NMR (400 MHz) spectrum of cycloaddition of DIBAC **10** and benzyl azide.

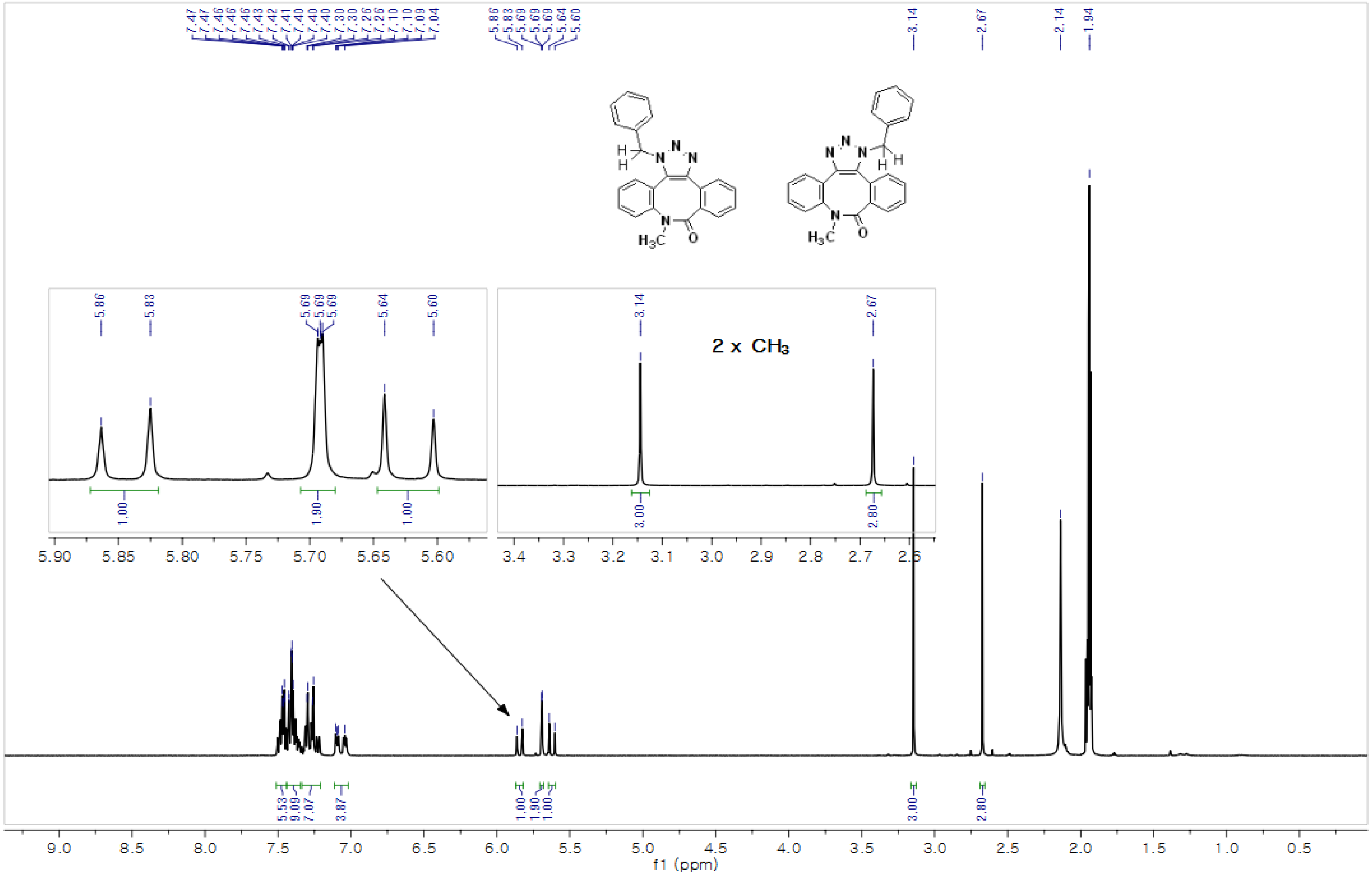

Figure S11. The ^{1}H NMR (400 MHz) spectrum of cycloaddition of BARAC **6** and benzyl azide .

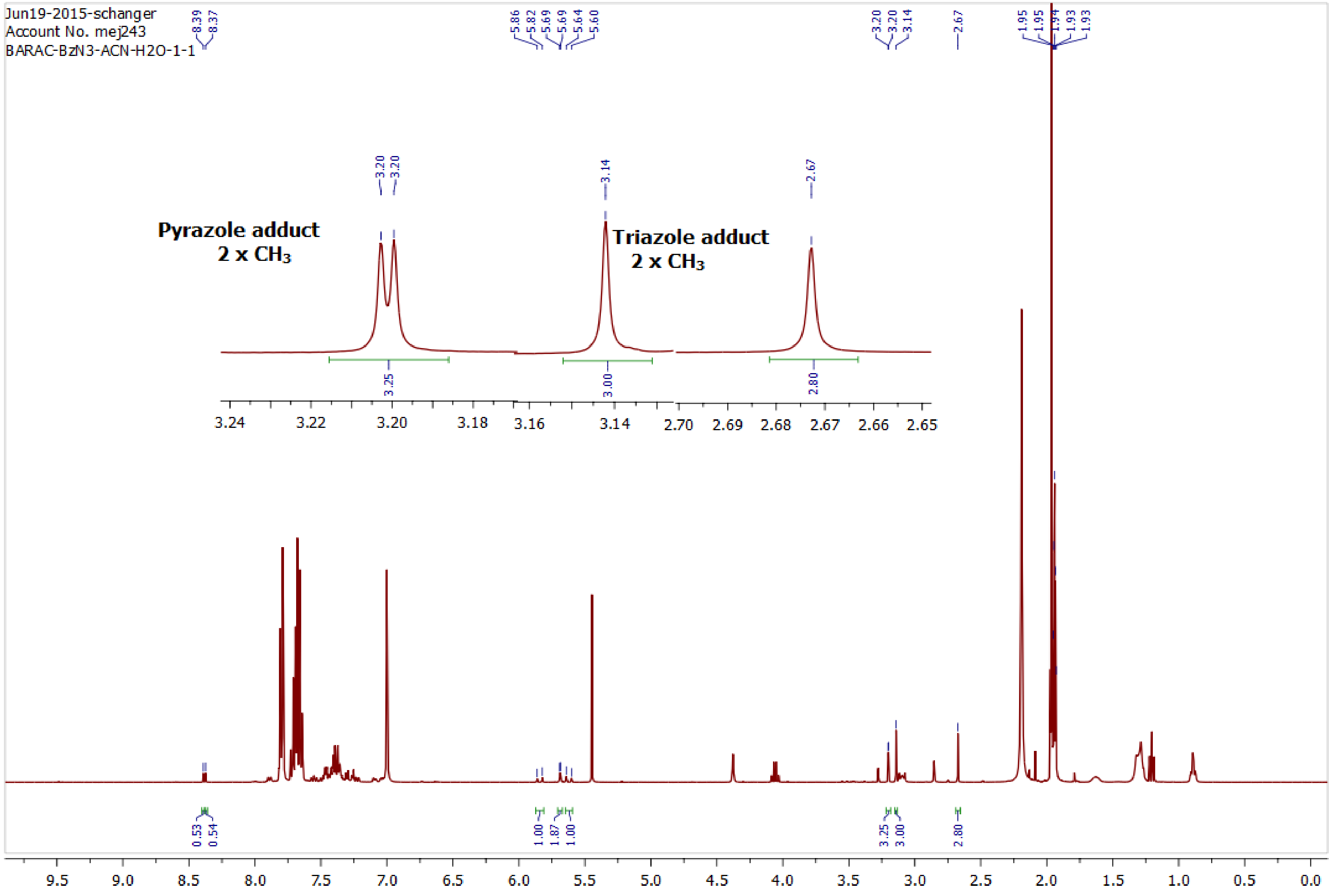

Figure S12. The ^{1}H NMR (400 MHz) competition experiment of BARAC **6** + benzyl azide + phenyl sydnone **1**.

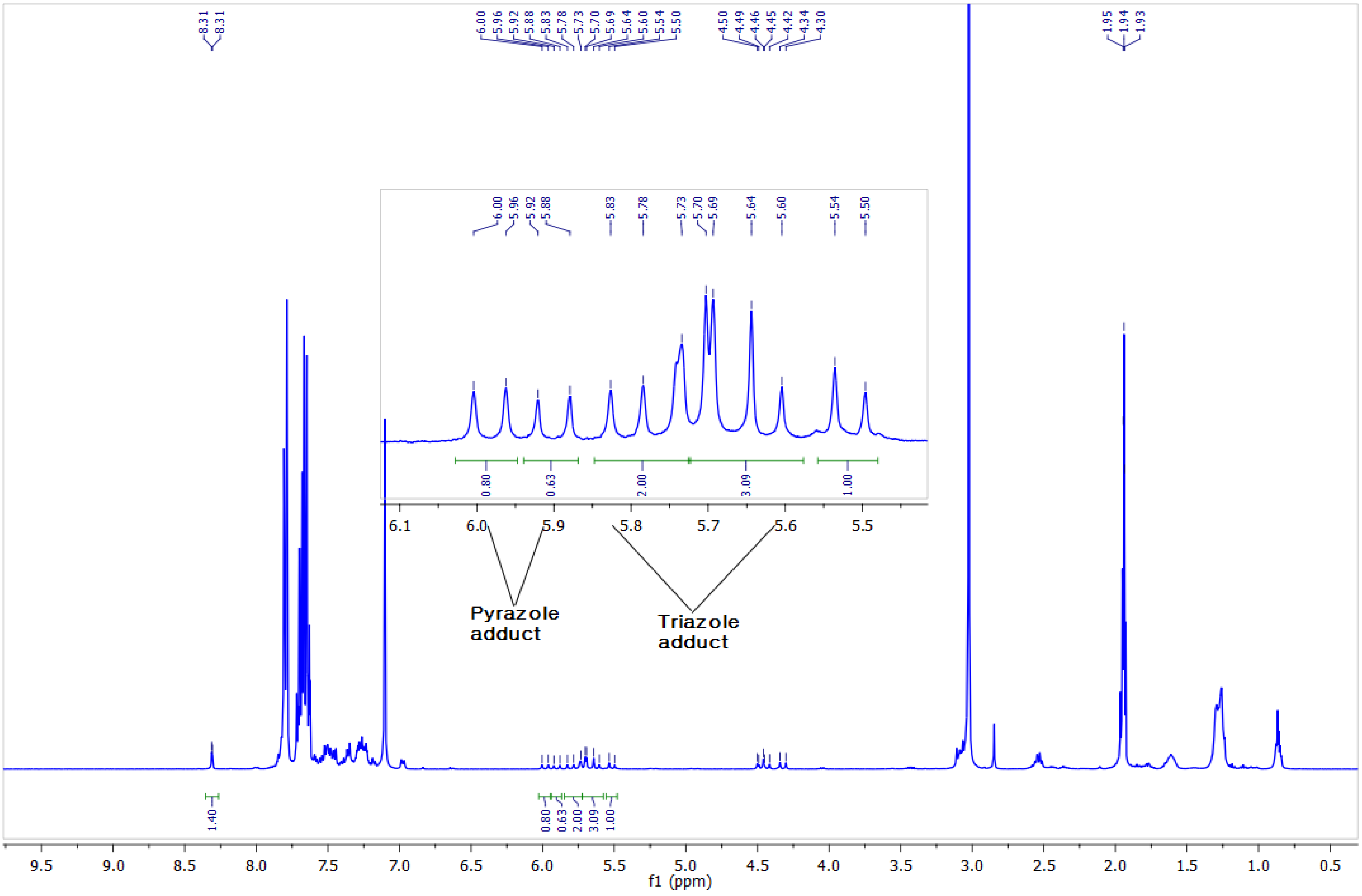

Figure S13. The ^{1}H NMR (400 MHz) competition experiment of DIBAC **10** + benzyl azide + phenyl sydnone **1**.

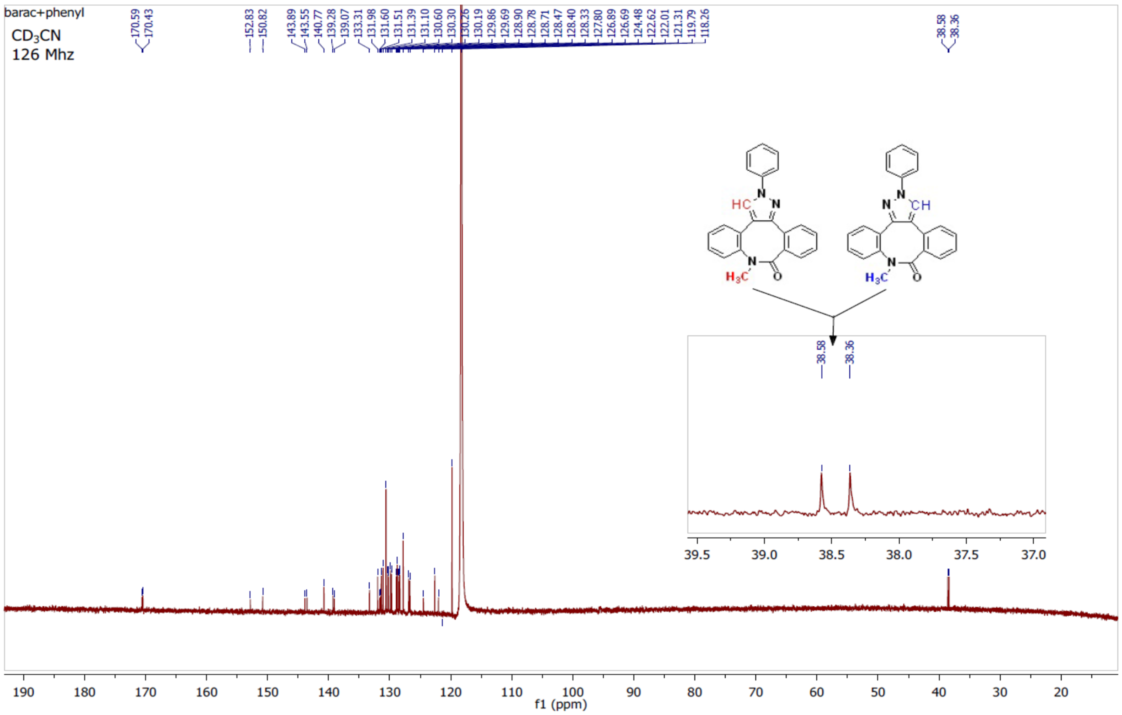

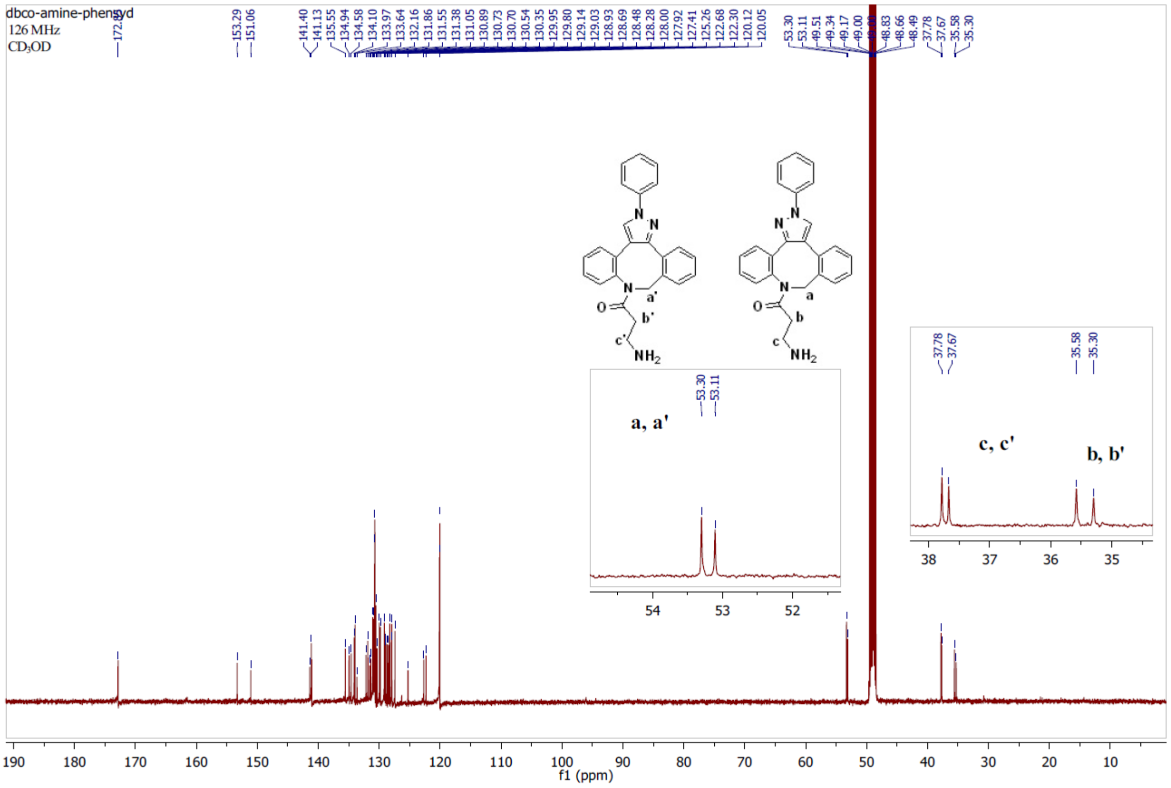

Figure S14. The ^{13}C NMR spectra of cycloadducts **7** and **11**.

References

(1) Frisch, M. J.; Trucks, G. W.; Schlegel, H. B.; Scuseria, G. E.; Robb, M. A.; Cheeseman, J. R.; Scalmani, G.; Barone, V.; Mennucci, B.; Petersson, G. A.; Nakatsuji, H.; Caricato, M.; Li, X.; Hratchian, H. P.; Izmaylov, A. F.; Bloino, J.; Zheng, G.; Sonnenberg, J. L.; Hada, M.; Ehara, M.; Toyota, K.; Fukuda, R.; Hasegawa, J.; Ishida, M.; Nakajima, T.; Honda, Y.; Kitao, O.; Nakai, H.; Vreven, T.; Montgomery, J. A., Jr.; Peralta, J. E.; Ogliaro, F.; Bearpark, M.; Heyd, J. J.; Brothers, E.; Kudin, K. N.; Staroverov, V. N.; Keith, T.; Kobayashi, R.; Normand, J.; Raghavachari, K.; Rendell, A.; Burant, J. C.; Iyengar, S. S.; Tomasi, J.; Cossi, M.; Rega, N.; Millam, J. M.; Klene, M.; Knox, J. E.; Cross, J. B.; Bakken, V.; Adamo, C.; Jaramillo, J.; Gomperts, R.; Stratmann, R. E.; Yazyev, O.; Austin, A. J.; Cammi, R.; Pomelli, C.; Ochterski, J. W.; Martin, R. L.; Morokuma, K.; Zakrzewski, V. G.; Voth, G. A.; Salvador, P.; Dannenberg, J. J.; Dapprich, S.; Daniels, A. D.; Farkas, O.; Foresman, J. B.; Ortiz, J. V.; Cioslowski, J.; Fox, D. J.; *Gaussian 09, revision D.01*; Gaussian Inc.: Wallingford, CT, 2013.

(2) (a) Zhao, Y.; Truhlar, D. G. *Theor. Chem. Acc.* **2008**, *120*, 215. (b) Zhao, Y.; Truhlar, D. G. *Acc. Chem. Res.* **2008**, *41*, 157.

(3) Hehre, W. J.; Radom, L.; Schleyer, P. v. R.; Pople, J. A. *Ab Initio Molecular Orbital Theory*, Wiley: New York, 1986.

(4) (a) Zhao, Y.; Truhlar, D. G. *Phys. Chem. Chem. Phys.* **2008**, *10*, 2813. (b) Ribeiro, R. F.; Marenich, A. V.; Cramer, C. J.; Truhlar, D. G. *J. Phys. Chem. B* **2011**, *115*, 14556.

(5) (a) Barone, V.; Cossi, M. *J. Phys. Chem. A* **1998**, *102*, 1995. (b) Cossi, M.; Rega, N.; Scalmani, G.; Barone, V. *J. Comput. Chem.* **2003**, *24*, 669. (c) Takano, Y.; Houk, K. N. *J. Chem. Theory Comput.* **2005**, *1*, 70.

(6) Wallace, S.; Chin, J. W. *Chem. Sci.* **2014**, *5*, 1742.

(7) Beckmann, H. S.; Niederwieser, A.; Wiessler, M.; Wittmann, V. *Chem. Eur. J.* **2012**, *18*, 6548.

(8) Gordon, C. G.; Mackey, J. L.; Jewett, J. C.; Sletten, E. M.; Houk, K. N.; Bertozzi, C. R. *J. Am. Chem. Soc.* **2012**, *134*, 9199.
